# Therapeutic Targeting of Decr1 Ameliorates Cardiomyopathy by Suppressing Mitochondrial Fatty Acid Oxidation in Diabetic Mice

**DOI:** 10.1002/jcsm.13761

**Published:** 2025-03-07

**Authors:** Qing‐Bo Lu, He‐Ting Sun, Kuo Zhou, Jia‐Bao Su, Xin‐Yu Meng, Guo Chen, Ao‐Yuan Zhang, An‐Jing Xu, Chen‐Yang Zhao, Yuan Zhang, Yao Wang, Hong‐Bo Qiu, Zhuo‐Lin Lv, Zheng‐Yang Bao, Jian Zhu, Feng Xiao, Xue‐Xue Zhu, Hai‐Jian Sun

**Affiliations:** ^1^ Department of Endocrinology Affiliated Hospital of Jiangnan University, Jiangnan University Wuxi Jiangsu China; ^2^ School of Pharmacy Collaborative Innovation Center of Advanced Drug Delivery System and Biotech Drugs in Universities of Shandong, Key Laboratory of Molecular Pharmacology and Drug Evaluation (Yantai University), Ministry of Education, Yantai University Yantai China; ^3^ Department of Cardiology The First Affiliated Hospital of Nanjing Medical University, Nanjing Medical University Nanjing China; ^4^ Department of Anesthesiology Affiliated Hospital of Jiangnan University, Jiangnan University Wuxi China; ^5^ MOE Medical Basic Research Innovation Center for Gut Microbiota and Chronic Diseases School of Medicine, Jiangnan University Wuxi China; ^6^ Research Institute for Reproductive Health and Genetic Diseases Wuxi Maternity and Child Health Care Hospital, Jiangnan University Wuxi China; ^7^ Department of Cardiology the Affiliated Wuxi People's Hospital of Nanjing Medical University, Wuxi People's Hospital, Nanjing Medical University Wuxi China; ^8^ State Key Laboratory of Natural Medicines China Pharmaceutical University Nanjing China

**Keywords:** cardiomyopathy, Decr1, diabetes, fatty acid oxidation, oxidative stress, PDK4

## Abstract

**Background:**

A significant increase in mitochondrial fatty acid oxidation (FAO) is now increasingly recognized as one of the metabolic alterations in diabetic cardiomyopathy (DCM). However, the molecular mechanisms underlying mitochondrial FAO impairment in DCM remain to be fully elucidated.

**Methods:**

A type 2 diabetes (T2D) mouse model was established by a combination of high‐fat diet (HFD) and streptozotocin (STZ) injection. Neonatal rat cardiomyocytes were treated with high glucose (HG) and palmitic acid (HP) to simulate diabetic cardiac injury. Gain‐ and loss‐of‐function approaches and RNA sequencing were utilized to investigate the role and mechanism of 2,4‐dienoyl‐CoA reductase 1 (Decr1) in DCM.

**Results:**

By integrating the genomic data available in the Gene Expression Omnibus (GEO) with DCM rodents, we found that the transcriptional level of Decr1 was consistently upregulated in DCM (+255% for diabetic heart, *p* < 0.0001; +281% for diabetic cells, *p* < 0.0001). Cardiomyocytes‐specific knockdown of Decr1 preserved cardiac function (+41% for EF, *p* < 0.0001; +24% for FS, *p* = 0.0052), inhibited cardiac hypertrophy (−34%, *p* < 0.0001), fibrosis (−69%, *p* < 0.0001), apoptosis (−56%, *p* < 0.0001) and oxidative damage (−59%, *p* < 0.0001) in DCM mice, while cardiomyocytes‐specific overexpression of Decr1 aggravated DCM (−28% for EF, *p* = 0.0347; −17% for FS, *p* = 0.0014). Deletion of Decr1 prevented high glucose/palmitate (HG/HP)‐induced hypertrophy (−22%, *p* = 0.0006), mitochondrial dysfunction and apoptosis (−74%, *p* < 0.0001) in cultured cardiomyocytes. Furthermore, RNA sequencing and functional analysis showed that Decr1 interacted with and upregulated pyruvate dehydrogenase kinase 4 (PDK4) in injured cardiomyocytes, and overexpression of PDK4 eliminated the benefits of Decr1 downregulation in DCM (−20% for EF, *p* = 0.0071; −28% for FS, *p* = 0.0022). Mechanistically, PDK4 acted as a kinase that induced phosphorylation and mitochondrial translocation of HDAC3. In the mitochondria, HDAC3 mediated the deacetylation of dehydrogenase trifunctional multienzyme complex α subunit (HADHA), contributing to excessive mitochondrial FAO and subsequent cardiac injury. From a screening of 256 natural products, we identified Atranorin and Kurarinone as potential inhibitors of Decr1, both demonstrating protective effects against DCM (Atranorin, +21% for EF, *p* = 0.0134; +24% for FS, *p* = 0.0006; Kurarinone, +20% for EF, *p* = 0.0183; +27% for FS, *p* = 0.0001).

**Conclusions:**

Our study delineates a molecular mechanism by which Decr1 potentiated higher mitochondrial lipid oxidation and cardiac damage by enhancing HADHA deacetylation through the PDK4/HDAC3 signalling pathway.

## 1 Introduction

Diabetes and its complications, including diabetic cardiomyopathy (DCM) have become a global epidemic, affecting approximately 463 million people worldwide, with projections suggesting a rise to 700 million cases by 2045 [1]. Clinical studies have shown that DCM is a leading cause of severe cardiovascular events and worsens the prognosis for diabetic patients [2]. The primary features of DCM include impaired left ventricular contraction, diastolic dysfunction, and ventricular hypertrophy, distinct from conditions such as hypertension and coronary syndrome [2]. Elevated glucose levels initiate a cascade of changes in cardiomyocytes, including disrupted calcium handling, mitochondrial dysfunction, myocardial interstitial fibrosis, cardiac autonomic neuropathy, and microvascular dysfunction, all contributing to the development and progression of DCM [3, 4]. Despite decades of research on the pathogenesis and clinical features of DCM in past decades, effective approaches to prevent and treat this disease remain limited.

Recently, mounting evidence suggests that metabolic disturbance plays a primary role in the pathogenesis of DCM by inducing mitochondrial dysfunction, oxidative stress, inflammation, and apoptosis [5, 6]. One of the significant metabolic alterations in diabetes is a marked increase in cardiac fatty acid oxidation (FAO) rates and the domination of fatty acids as the major energy source in the heart [7]. Impaired glucose oxidation in diabetic hearts is partially due to reduced cardiac insulin signalling, leading to increased reliance on FAO for energy [8]. This shift from glucose to FAO creates an imbalance in oxidative metabolism, which is believed to contribute to mitochondrial dysfunction and is often linked to cardiac dysfunction [8, 9]. The increased FAO elevates mitochondrial reactive oxygen species (ROS) production, leading to mitochondrial dysfunction and cell apoptosis, which may exacerbate DCM [9, 10]. Thereby, inhibiting excessive mitochondrial FAO may be a viable strategy for the prevention and treatment of DCM.

To explore potential mechanisms contributing to DCM in the present study, we analysed published genomic data from a murine model of DCM using the Gene Expression Omnibus (GEO) databases. Decr1 (2,4‐dienoyl‐CoA reductase 1) was found to be significantly elevated in DCM mice across the four GEO databases, emphasizing its importance in DCM pathogenesis. Consistently, the protein and mRNA expression levels of Decr1 were strikingly upregulated in the heart of diabetic rodents. By coincidence, Decr1 is an essential enzyme in the mitochondrial β‐oxidation pathway of unsaturated fatty acids since it plays a crucial role in metabolizing polyunsaturated fatty acids with conjugated double bonds, converting them into intermediates that can be further processed by other enzymes in the β‐oxidation pathway. Thus, we speculated that Decr1 might have an important impact on DCM. To investigate this possibility, we examined the effects of Decr1 on DCM in mice and neonatal rat cardiomyocytes incubated with a high glucose combined with palmitic acid (HG/HP) using gain‐ and loss‐of‐function approaches. RNA sequencing, bioinformatics analysis, detection of FAO and glucose oxidation, co‐immunoprecipitation, and drug screening were used to explore the molecular mechanisms underlying the role of Decr1 in DCM pathogenesis. Using these strategies, we found that cardiac Decr1 expression was upregulated in both DCM mice and cardiomyocytes exposed to HG/HP. In vivo and in vitro studies disclosed that deletion of Decr1 in cardiomyocytes alleviated cardiac abnormalities in diabetes, while overexpression of Decr1 exacerbated DCM. Mechanistically, Decr1 interacted with and upregulated PDK4, leading to the phosphorylation and mitochondrial translocation of HDAC3. After that, HDAC3 promoted HADHA deacetylation, which in turn evoked mitochondrial FAO and myocardial injury in DCM. Moreover, our drug screening identified Atranorin and Kurarinone as compounds that ameliorated myocardial injury in DCM by binding to and inhibiting Decr1. In summary, these findings highlight the significant role of cardiac Decr1 in regulating hyperglycaemia‐induced myocardial damage.

## 2 Materials and Methods

### 2.1 Animals

All animal experimental procedures were conducted in accordance with the Guide for the Care and Use of Laboratory Animals (8th edition, revised 2011), as outlined by the US National Institutes of Health. Approval for the study was granted by the Laboratory Animal Welfare & Ethics Committee of Jiangnan University.

### 2.2 Cell Culture

Neonatal rat cardiomyocytes were isolated from 1‐ to 3‐day‐old Sprague–Dawley rats, and cardiac endothelial cells and fibroblasts from mice were cultured as we previously described [11, 12].

### 2.3 Others

All details of our methods are shown in Data S1.

## 3 Results

### 3.1 Decr1 Expression Was Upregulated in DCM Mice and High Glucose/Palmitate (HG/HP)‐exposed Cardiomyocytes

**FIGURE 1:**
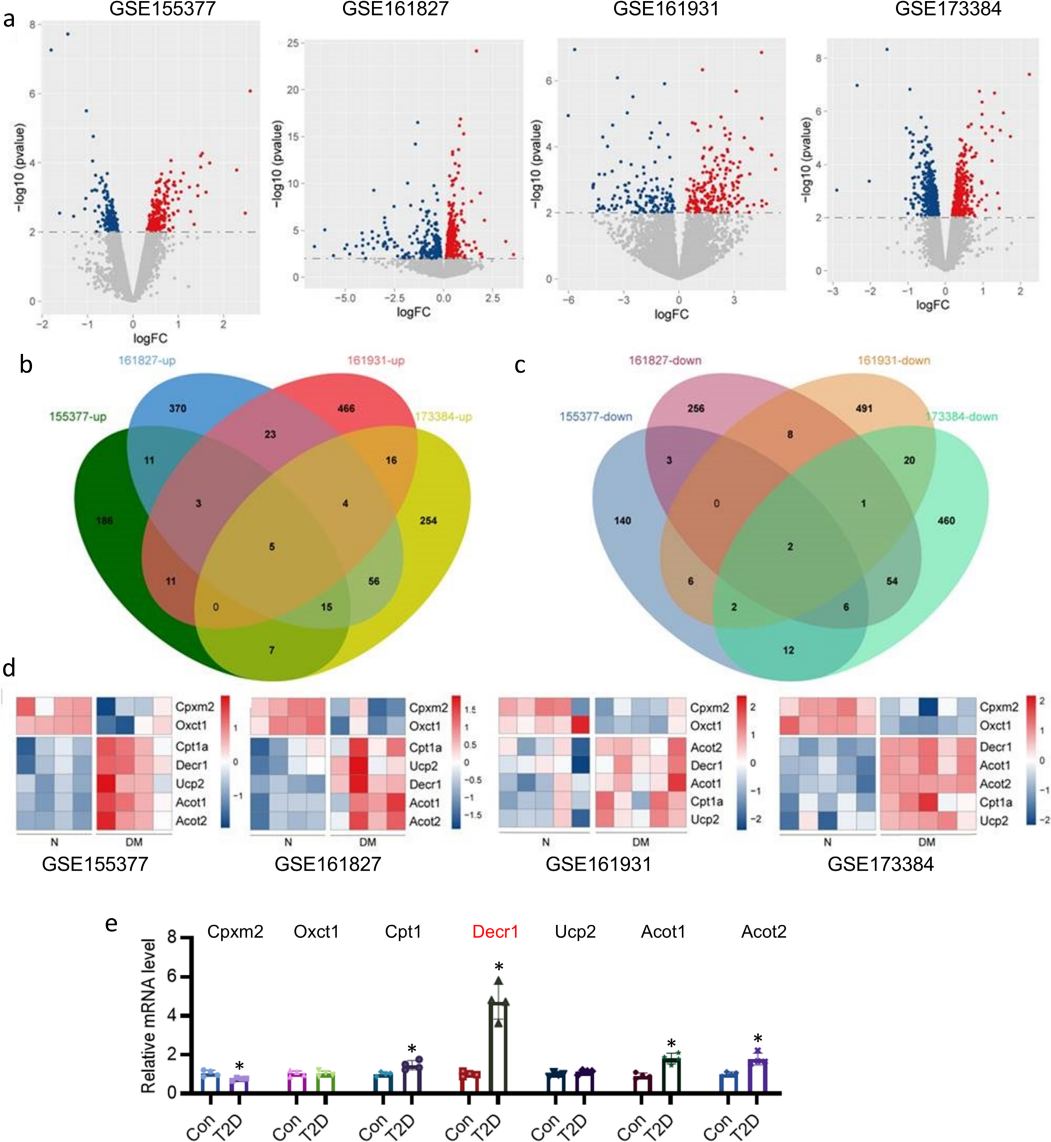
The transcriptional level of Decr1 is upregulated in DCM. (a) Volcano plot showing the differentially expressed genes from GSE155377 database, GSE161827 database, GSE161931 database, and GSE173384 database. (b) Vein diagram showing the upregulated genes from GSE155377 database, GSE161827 database, GSE161931 database, and GSE173384 database. (c) Vein diagram showing the downregulated genes from GSE155377 database, GSE161827 database, GSE161931 database, and GSE173384 database. (d) Seven genes upregulated and downregulated in all four databases. (e) Relative mRNA level of Cpxm2, Oxct1, Cpt1, Decr1, Ucp2, Acot1, and Acot2 in the heart from control mice and T2D mice. Data were calculated as means ± SD. *p < 0.05 vs. Control (Con).

To examine the gene expression profiles associated with DCM, we conducted a comprehensive analysis of the publicly available genomic data from the GEO database, including GSE155377, GSE161827, GSE161931 and GSE173384. Both up‐ and down‐regulated genes were identified between normal and DCM heart in these datasets (Figure 1a). Then, the intersection of significantly upregulated and downregulated genes in four databases was analysed through vein analysis (Figure 1b,c). Seven genes with consistent pattern changes across four databases were discovered by intersection analysis, including Cpxm2, Oxct1, Cpt1α, Decr1, Ucp2, Acot1, Acot2 (Figure 1d). In line with the results obtained by mining the GEO database, the mRNA levels of Cpt1α, Decr1, Acot1, and Acot2 were significantly upregulated in the heart of T2D mice (Figure 1e), while Decr1 caught our attention since its mRNA levels showed the most significant changes. Notably, an online database, named HUMAN PROTEIN ATLAS (https://www.proteinatlas.org/) aiming at mapping all human proteins in cells, tissues, and organs, revealed that Decr1 is expressed in all organs throughout the body, with its expression being most pronounced in the cardiomyocytes (Figure S1a). Then, we compared the differences in Decr1 expression levels in different cells of human heart, and found that Decr1 displayed the highest expression in cardiomyocytes (Figure S1b,c). Also, the online database of HUMAN PROTEIN ATLAS showed the immunohistochemical staining of Decr1 in the heart tissues (Figure S1d). Moreover, single cell sequencing of mouse heart tissues also demonstrated that Decr1 is mainly expressed in cardiomyocytes using the Tabula Muris database (Figure S1e). As expected, the translational and transcriptional levels of Decr1 were significantly higher in heart tissues from DCM mice (Figure S2a). In addition, we found the abundance of Decr1 was higher in cardiac muscle in T2D mice, as evidenced by immunohistochemistry (Figure S2b) and immunofluorescence (Figure S2c). Interestingly, the mRNA level of Decr1 was only elevated in isolated cardiomyocytes, instead of cardiac fibroblasts and endothelial cells (Figure S2d). Upon exposure of high glucose/palmitate (HG/HP), the protein and mRNA levels of Decr1 were notably increased in primary neonatal rat cardiomyocytes (Figure S2e,f).

### 3.2 Decr1 Deletion Ameliorated, Whereas Decr1 Overexpression Aggravated Cardiac Dysfunction and Remodelling in Diabetic Mice

**FIGURE 2:**
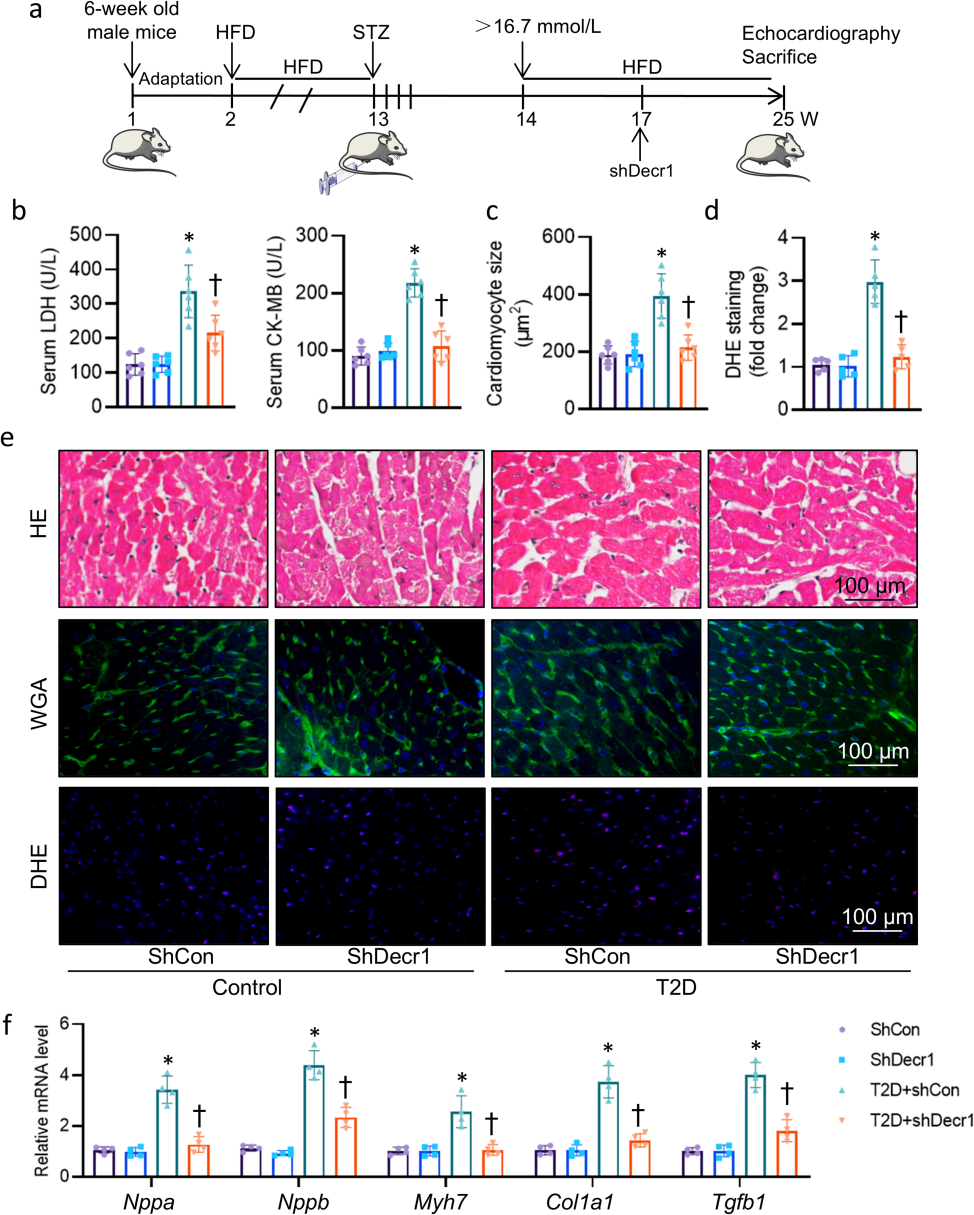
Cardiac‐specific knockdown of Decr1 ameliorates DCM in T2D mice. (a) The flow chart of animal experiments. (b) Serum LDH and CK‐MB level. (c) Cardiomyocyte size measured by WGA staining. At least 100 cells measured in different visual fields from 6 samples per group. Each point on each column represents the average area of 100 myocardial cells in each sample. The quantification of average cross‐sectional area of cardiomyocytes compared with control in the indicated groups was shown in the bar graph. (d) The quantification of DHE staining. (e) Representative images of H&E staining, WGA staining, and DHE staining of left ventricle myocardium. (f) Relative mRNA level of *Nppa*, *Nppb*, *Myh7*, *Col1a1*, and *Tgfb1*. Data were calculated as means ± SD. *p < 0.05 vs. ShCon. †*p* < 0.05 vs. T2D + ShCon.

Upregulation of Decr1 in diabetic hearts propelled us to investigate whether or not cardiac‐specific knockdown of Decr1 could ameliorate diabetes‐related cardiac pathologies (Figure 2a). After tail vein injection of AAV9 vectors carrying Decr1 shRNA under the control of a cTnT promoter, the protein expression of Decr1 was markedly downregulated in isolated cardiomyocytes (Figure S3a). Our results demonstrated that cardiac‐specific knockdown of Decr1 did not change the body weight, fasting blood glucose (FBG) levels, insulin levels, total cholesterol (TC), or triacylglycerol (TG) in T2D mice (Table S5). Nonetheless, deficiency of Decr1 decreased heart weight (HW)/body weight (BW) ratio, HW/tibial length ratio (Table S5), and retarded serum levels of cardiac damage markers, such as lactate dehydrogenase (LDH) and creatine kinase‐cardiac (CK‐MB) (Figure 2b). Cardiac‐specific downregulation of Decr1 improved T2D‐induced cardiac dysfunction, as evidenced by significant increases in left ventricular ejection fraction (EF), and fractional shortening (FS) (Figure S3b, Table S5). Hyperglycemia led to cardiomyocyte enlargement in diabetic mouse heart, as observed in haematoxylin and eosin (H&E) and WGA staining, but these changes were notably reversed by deficiency of Decr1 (Figure 2c,e). Assessment of cardiac fibrosis in mice using Sirius Red staining showed that downregulation of Decr1 afforded a protection against diabetes‐evoked myocardial fibrosis (Figure S3c,d). The apoptosis in the Decr1‐deficient heart did not show the same increase as seen in un‐treated T2D mice (Figure S3c,e). Analysis of the harvested heart tissues showed higher oxidative stress in T2D mice but not mice with cardiac deficiency of Decr1, as indicated by DHE staining (Figure 2d,e). Similarly, cardiac‐specific knockdown of Decr1 blocked the upregulated mRNA levels of hypertrophic and fibrotic genes in T2D mice (Figure 2f). In assays of mitochondrial oxidative phosphorylation (OXPHOS)‐complex activity, complexes I, II, III, and IV activities were reduced in DCM mice, such changes were reversed by cardiac deficiency of Decr1 (Figure S3f). Accordingly, the lower ATP levels were observed in the heart of DCM mice, but this was restored by downregulation of Decr1 in the heart (Figure S3g). As such, Decr1 deficiency alleviated myocardial injury and mitochondrial damage in T2D mice.

By contrast to what we observed in T2D mice with ablation of Decr1, cardiac‐specific overexpression of Decr1 exhibited the opposite effects (Figure S4a). Ectopic overexpression resulted in increased Decr1 protein by nearly 4‐fold in the heart (Figure S4b). Overexpression of Decr1 had no effect on the body weight gain, FBG, and lipid levels in mice (Table S6). Serum levels of LDH and CK‐MB were dramatically upregulated in T2D mice injected with Decr1 overexpression when compared with empty vectors‐injected mice (Figure S4c). We also uncovered that cardiac‐specific overexpression of Decr1 further reduced cardiac function in diabetic mice (Figure S4d, Table S6). HE and WGA staining revealed that Decr1‐overexpressed mice exhibited exacerbated cardiomyocyte area caused by diabetes (Figure S4e,f). Notably, the levels of cardiac fibrosis (Figure S4e,g), apoptosis (Figure S4e,h), and oxidative stress (Figure S4e,i) were more profound in T2D mice with Decr1 overexpression compared to those in T2D mice. The pro‐hypertrophic and pro‐fibrotic effects of Decr1 overexpression were further supported by increased mRNA levels of *Nppa*, *Nppb*, *Myh7*, *Col1a1*, and *Tgfb1* in diabetic heart (Figure S4j). The activities of mitochondrial complexes I, II, III and IV were significantly reduced in the hearts of DCM mice. These deficits were further worsened by the cardiac‐specific overexpression of Decr1 (Figure S5a). Consistent with these findings, ATP levels were notably lower in the heart of DCM mice, and overexpression of Decr1 further reduced ATP level in diabetic heart (Figure S5b). Thereby, cardiac injuries were enhanced in diabetic mice with cardiac‐specific overexpression of Decr1.

### 3.3 Decr1 Silencing Suppressed, Whereas Decr1 Overexpression Worsened HG/HP‐Induced Injury in Cardiomyocytes

**FIGURE 3:**
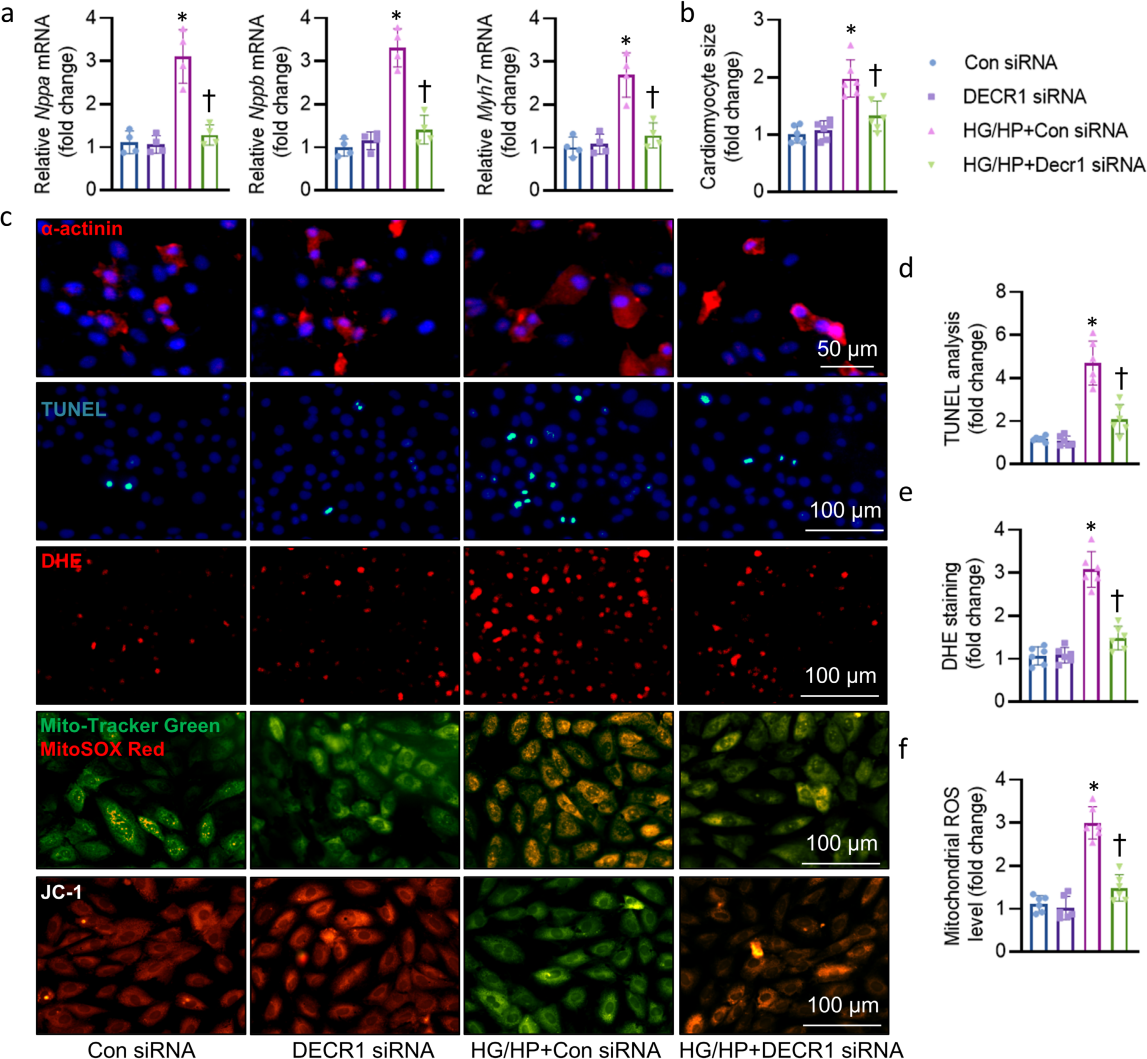
Downregulation of Decr1 protects cardiomyocytes against HG/HP‐induced injury. (a) Relative mRNA level of *Nppa*, *Nppb*, *Myh7*. (b) Cardiomyocyte size measured by α‐actinin staining. (c) Representative images of α‐actinin staining, TUNEL staining, DHE staining, mitochondrial ROS levels, and JC‐1 staining. (d) The quantification of TUNEL staining. (e) The quantification of DHE staining. (f) The quantification of mitochondrial ROS levels. Data were calculated as means ± SD. *p < 0.05 vs. Con siRNA. †*p* < 0.05 vs. HG/HP + Con siRNA.

Based on the in vivo results of Decr1 manipulation in DCM mice, we wanted to examine whether such effects were replicated in cultured cardiomyocytes exposed to HG/HP. We first examined whether Decr1 downregulation was able to suppress the deleterious effects of HG/HP on cardiomyocytes. Transfection of Decr1 siRNA significantly downregulated the protein and mRNA levels of Decr1 (Figure S6a–c), and obviously inhibited HG/HP‐induced LDH release in primary cardiomyocytes (Figure S7). RT‐PCR showed that the mRNA levels of hypertrophic genes, *Nppa*, *Nppb,* and *Myh7* were enhanced in cardiomyocytes challenged by HG/HP, an effect that was normalized by knockdown of Decr1 (Figure 3a). It was found that HG/HP exposure enlarged cellular size in cardiomyocytes, as indicated by α‐actinin staining, while this was prevented by silencing Decr1 (Figure 3b,c). TUNEL analysis showed that downregulation of Decr1, which itself had no obvious effect, significantly reduced HG/HP‐induced cell apoptosis (Figure 3c,d). DHE staining was used to detect ROS levels in cardiomyocytes. HG/HP stimulation increased intracellular ROS generation and this effect was attenuated by silencing Decr1 (Figure 3c,e). We further examined mitochondrial ROS generation with MitoSOX™ Red staining. As shown in Figure 3c, HG/HP remarkably promoted mitochondrial ROS generation in the mitochondria, and this effect was reversed by depletion of Decr1. The mitochondrial permeability transition, an important step in the induction of cellular apoptosis, was also determined using the unique fluorescent cationic dye, JC‐1. Results showed that HG/HP induced loss of red JC‐1 aggregate fluorescence, and higher green monomer fluorescence was detected in the cytoplasm (Figure 3c). This effect was also reversed by knockdown of Decr1 (Figure 3c). In sharp contrast, overexpression of Decr1 further deteriorated HG/HP‐induced cardiomyocyte injury (Figure S6d–f, S8).

### 3.4 RNA Sequencing Revealed That Decr1 Upregulated PDK4 in Cardiomyocytes

**FIGURE 4:**
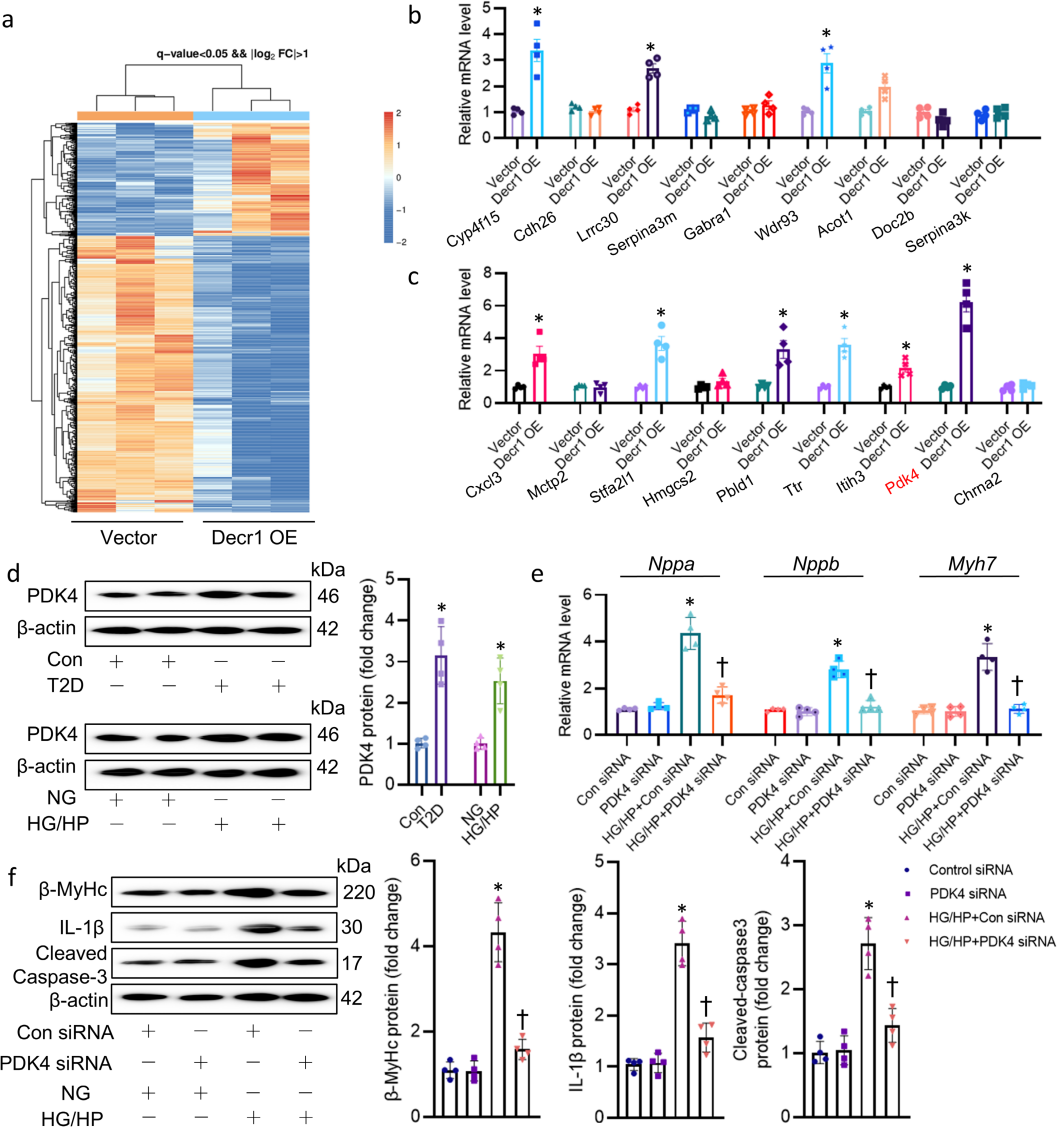
RNA sequencing revealed that Decr1 upregulated PDK4 in cardiomyocytes. (a) Heatmap showing the differentially expressed genes in the heart after Decr1 overexpression. (b, c) Validation of top 18 upregulated gene mRNA level. (d) The protein expression of Decr1 in diabetic heart and HG/HP‐incubated cardiomyocytes. (e) Relative mRNA level of *Nppa*, *Nppb*, *Myh7* in cardiomyocytes after knockdown of PDK4. (f) The protein expression of β‐MyHc, IL‐1β, cleaved caspase‐3 in cardiomyocytes after knockdown of PDK4. Data were calculated as means ± SD. *p < 0.05 vs. Vector or Con siRNA. †*p* < 0.05 vs. HG/HP + Con siRNA.

Next, we carried out RNA sequencing to explore the transcriptomic changes caused by Decr1 overexpression. The cluster analysis of differential gene expression level showed that 235 genes were upregulated and 574 genes were downregulated after Decr1 overexpression (Figure 4a). We then conducted RT‐PCR to examine the mRNA levels of the top 18 elevated genes, and found that Decr1 overexpression markedly upregulated the mRNA level of PDK4 (Figure 4b,c). Consistent with this, the protein expression of PDK4 was highly expressed in diabetic heart and HG/HP‐exposed cardiomyocytes (Figure 4d). Western blotting analysis demonstrated increased protein expression of PDK4 in Decr1‐overexpressed cardiomyocytes under both NG and HG/HP conditions (Figure S9a–c). By contrast, silencing Decr1 not only suppressed the protein of PDK4, but also prevented HG/HP‐induced expression of PDK4, indicating that Decr1 may be an upstream mediator of PDK4 (Figure S9d–f). To further investigate the functional role of PDK4 in DCM, we used PDK4 siRNA to downregulate this protein (Figure S10a). As anticipated, PDK4 downregulation rendered cardiomyocytes more invulnerable to HG/HP‐induced hypertrophy, as seen by lower mRNA levels of *Nppa*, *Nppb,* and *Myh7* (Figure 4e). Additionally, downregulation of PDK4 curbed the protein expression of β‐MyHc, IL‐1β, and cleaved caspase‐3 in HG/HP‐incubated cardiomyocytes, suggesting that PDK4 may be a driver for diabetes‐induced cardiac hypertrophy, apoptosis and oxidative damage (Figure 4f). Overall, Decr1 may be a positive regulator of PDK4, leading to hyperglycemia‐induced cardiomyocyte injury.

### 3.5 Overexpression of PDK4 Eliminated the Benefits of Decr1 Deficiency in DCM

**FIGURE 5:**
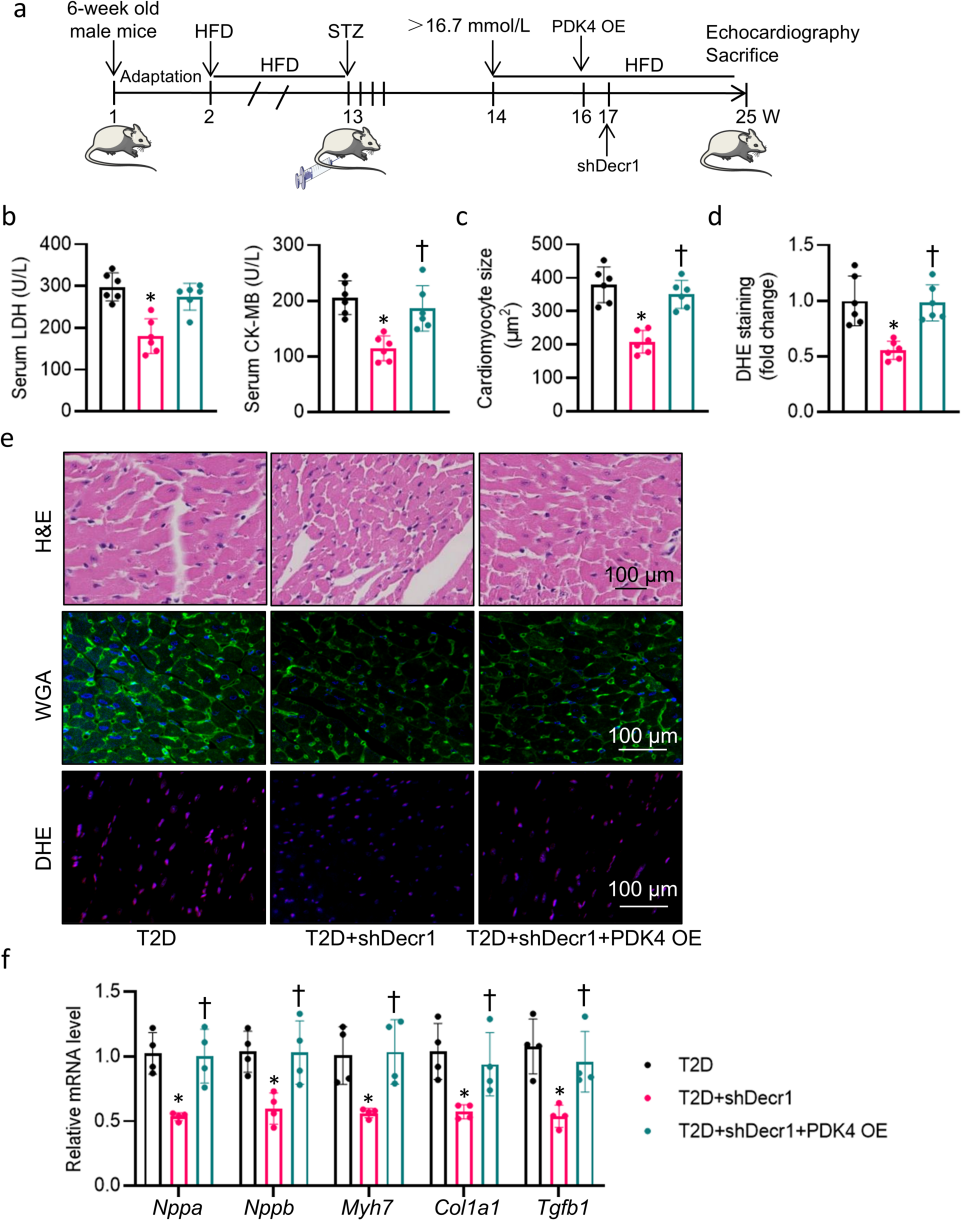
Overexpression of PDK4 eliminated the benefits of Decr1 deficiency in DCM. (a) The flow chart of animal experiments. (b) Serum LDH level and CK‐MB level. (c) Cardiomyocyte size measured by WGA staining. At least 100 cells measured in different visual fields from six samples per group. Each point on each column represents the average area of 100 myocardial cells in each sample. The quantification of average cross‐sectional area of cardiomyocytes compared with control in the indicated groups was shown in the bar graph. (d) The quantification of DHE staining. (e) Representative images of H&E staining, WGA staining, and DHE staining of left ventricle myocardium. (f) Relative mRNA level of *Nppa*, *Nppb*, *Myh7*, *Col1a1*, and *Tgfb1*. Data were calculated as means ± SD. *p < 0.05 vs. T2D. †*p* < 0.05 vs. T2D + ShDecr1.

Next, it is interesting to know whether overexpression of PDK4 blunted the protection of Decr1 deficiency against DCM in vivo (Figure 5a). Neither PDK4 overexpression nor Decr1 downregulation affected the body weight, FBG, and lipid profiles in T2D mice (Table S7). As mentioned earlier, downregulation of Decr1 diminished the serum levels of LDH and CK‐MB in T2D mice, while this effect was reversed by overexpression of PDK4 (Figure 5b). Assessment of heart function revealed that Decr1 deficiency‐mediated improvement of cardiac function was largely compromised in T2D mice with PDK4 overexpression (Figure S11a). Likewise, reduced cardiomyocyte hypertrophy, inhibited cardiac fibrosis, apoptosis, and oxidative stress were seen in heart tissues of diabetic mice with Decr1 deficiency as compared to untreated diabetic mice (Figure 5c–e, S11b–d). However, such changes were counteracted by PDK4‐overexpressed T2D mice (Figure 5c–e, S11b–d). These alterations were also confirmed by gene marker levels of hypertrophy and fibrosis (Figure 5f). Overall, these findings suggest that cardiac‐specific downregulation of Decr1 relieved DCM by targeting and suppressing PDK4.

### 3.6 Interaction of Decr1 With PDK4 Accelerated Lipid Oxidation and Reduced Glucose Oxidation in DCM

**FIGURE 6:**
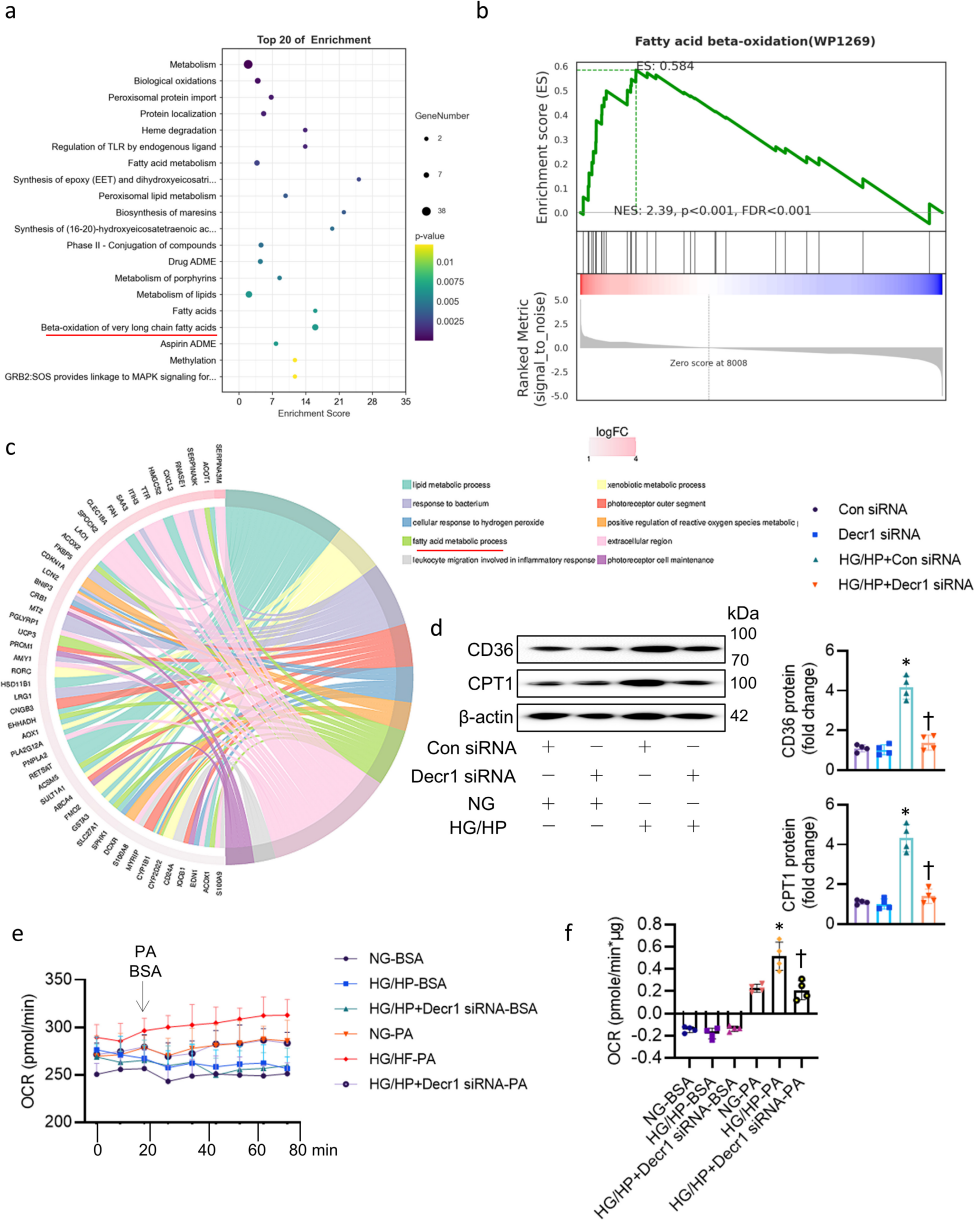
Interaction of Decr1 with PDK4 accelerated lipid oxidation and reduced glucose oxidation in DCM. (a) KEGG analysis of the differentially expressed genes in the heart after Decr1 overexpression. (b) GSEA analysis. (c) Helin diagram analysis. Ten significantly enriched GO entries and corresponding differentially expressed genes can be seen. The plot is divided into left and right sides, with the left side showing the top 10 genes with the highest|logFC|in each category of each GO entry, and the right side showing the 10 GO entries with the lowest *p*‐value or q‐value selected. The middle line represents the correspondence between genes and entries. The outer heatmap represents the logFC value of the corresponding gene. (d) The protein expression of CD36 and CPT1. (e) Kinetic OCR responses of isolated cardiomyocytes to palmitate acid. (f) Calculated palmitate acid oxidation. Palmitate acid oxidation is calculated by the OCR increase by normalizing cell protein contents. Data were calculated as means ± SD. *p < 0.05 vs. Con siRNA or NG‐PA. †*p* < 0.05 vs. HG/HP + Con siRNA or HG/HP‐PA.

In light of the regulatory role of Decr1 in PDK4 expression, we then set out to investigate whether Decr1 exerted its actions on DCM by interacting with PDK. Importantly, both proteins are highly expressed in the mitochondrion. We showed the direct binding of Decr1 to PDK4 using the immunoprecipitation (IP) and glutathione S‐transferase (GST) pulldown assay (Figure S12a–c). To map the Decr1‐interacting region of PDK4, a series of Decr1 and PDK4 truncated mutants were constructed. We found that the intermediate region (335–411) of PDK4 interacted with the intermediate region (151–335) of Decr1 (Figure S12d). Confocal microscopy further confirmed the interaction between these two proteins (Figure S12e). Moreover, the complex of Decr1/PDK4 was enhanced in cardiomyocytes upon exposure of HG/HP (Figure S12f). As is known, PDK4 plays a crucial role in regulating fatty acid oxidation by modulating the activity of the pyruvate dehydrogenase complex (PDC). PDC is a key enzyme that controls the conversion of pyruvate, a product of glycolysis, into acetyl‐CoA, which then enters the tricarboxylic acid (TCA) cycle. PDK4 phosphorylates and inactivates PDC, thus inhibiting the conversion of pyruvate to acetyl‐CoA and promoting a metabolic shift towards FAO over glucose oxidation. Thus, it is likely that Decr1 might act on PDK4 to regulate FAO in DCM. It is worth noting that Decr1 directly affected the pathway of FAO in the heart, using KEGG analysis (Figure 6a), Gene Set Enrichment Analysis (GSEA) (Figure 6b), and Helin diagram analysis (Figure 6c). CD36, a fatty acid transporter, is essential for fatty acid uptake in cardiac myocytes, while carnitine palmitoyltransferase 1 (CPT1) controls the entry of long‐chain fatty acids into mitochondria for oxidation. Both enzymes are crucial for cardiac fatty acid metabolism. In the present study, we found higher protein expression of CD36 and CPT1 levels in HG/HP‐incubated cardiomyocytes (Figure 6d). However, silencing Decr1 reversed the increases in both CD36 and CPT1 proteins (Figure 6d). Palmitate conjugated with BSA was used as a substrate for fatty acid oxidation, with BSA alone as the control. As shown in Figure 6e, the addition of palmitate‐BSA (1 mM) significantly increased the oxygen consumption rate (OCR) in diabetic cardiomyocytes compared to control cardiomyocytes. Knockdown of Decr1 treatment reduced this increase. The quantification in Figure 6e confirmed a higher palmitate oxidation rate in diabetic cardiomyocytes, which was significantly reduced by deficiency of Decr1 (Figure 6f). Pyruvate dehydrogenase (PDH) is a rate‐limiting enzyme that catalyses the conversion of pyruvate to acyl‐CoA, thereby promoting glucose oxidation. PDK4, the predominant isoform of PDKs in the heart, induces S256 phosphorylation of the E1 subunit of the PDH complex, leading to PDH deactivation [7]. In HG/HP‐injured cardiomyocytes, the phosphorylation level of PDH was significantly elevated (Figure S13a). However, deletion of Decr1 significantly reduced phosphorylated PDH level (Figure S13a). Glucose addition triggered a significant increase in oxygen consumption rate (OCR) in normal cardiomyocytes, but only a minor increase in diabetic cardiomyocytes (Figure S13b). Notably, Decr1 knockdown enhanced glucose‐stimulated OCR (Figure S13b). The pattern observed for pyruvate oxidation mirrored that of glucose oxidation (Figure S13c). Collectively, these findings suggest that downregulation of Decr1 can restore the reduced glucose and pyruvate oxidation rates in diabetic heart.

### 3.7 PDK4 Induced Phosphorylation and Mitochondrial Translocation of HDAC3 That Led to HADHA Deacetylation

**FIGURE 7:**
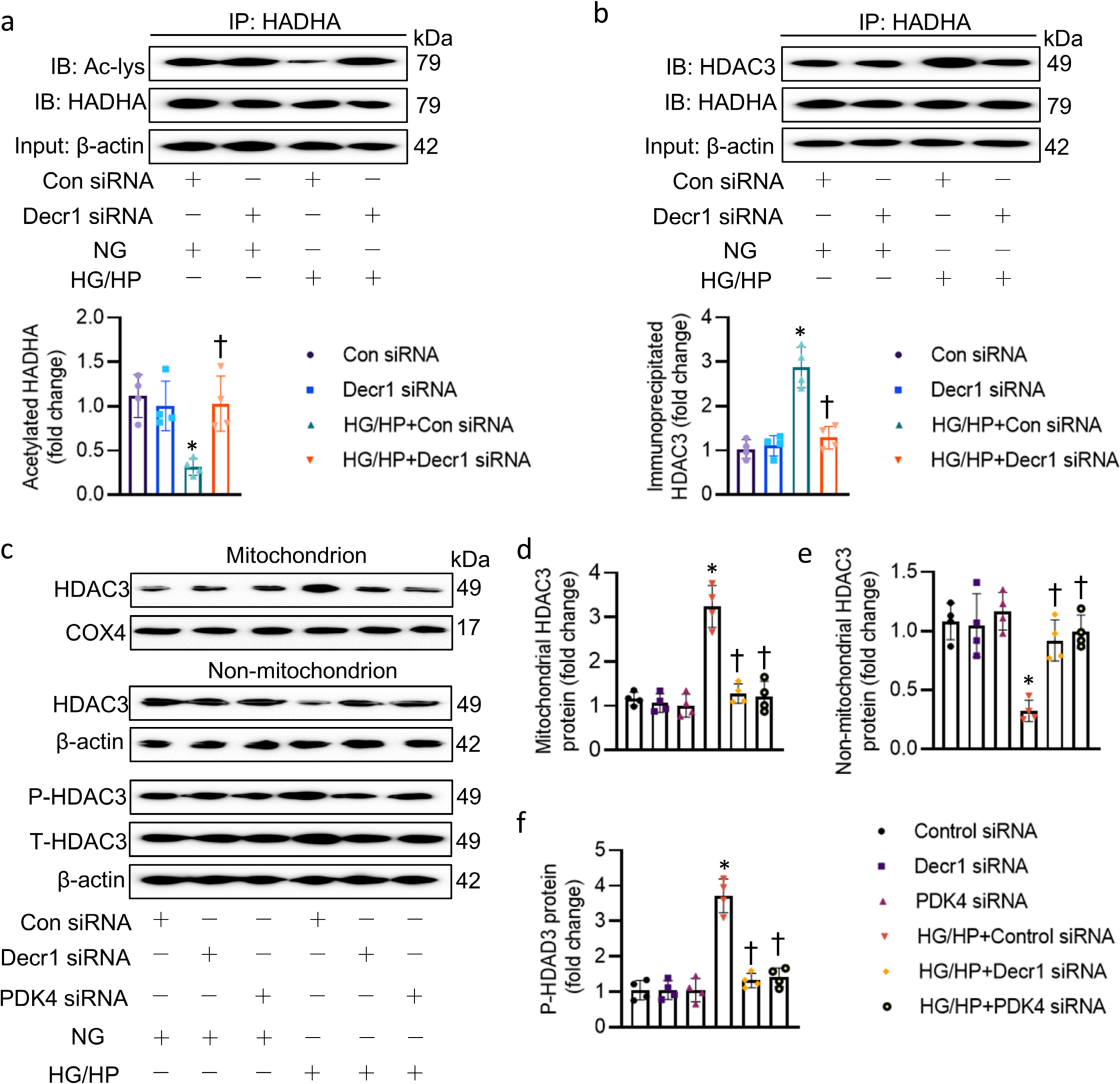
PDK4 induced phosphorylation and mitochondrial translocation of HDAC3 that led to HADHA deacetylation. (a) HADHA deacetylation level in cardiomyocytes after Decr1. (b) Interaction of HADHA with HDAC3 in cardiomyocytes after Decr1. (c–f) The protein of HDAC3 in the mitochondria and non‐mitochondria, as well as HDAC3 phosphorylation after knockdown of Decr1 or PDK4. Data were calculated as means ± SD. *p < 0.05 vs. Con siRNA. †*p* < 0.05 vs. HG/HP + Con siRNA.

HADHA (hydroxyacyl‐CoA dehydrogenase trifunctional multienzyme complex subunit α) is a critical enzyme involved in mitochondrial FAO. The deacetylation of HADHA, mediated by enzymes like histone deacetylase 3 (HDAC3), can significantly impact its activity and the overall efficiency of FAO [13]. Deacetylation typically enhances the enzymatic function of HADHA, leading to impaired FAO, contributing to mitochondrial dysfunction and associated metabolic disturbances [13]. The direct phosphorylation of HDAC3 at Ser‐424 is able to enhance its HDAC activity [14]. Of note, PDK4 functions as a kinase that promotes vascular calcification by phosphorylating SMAD1/5/8 via direct interaction, which enhances BMP2 signalling [15]. Based on these previous findings, we hypothesized that PDK4‐induced phosphorylation of HDAC3 might enhance its HDAC activity, leading to HADHA deacetylation and subsequent impaired mitochondrial FAO. As expected, HADHA acetylation levels were significantly reduced in HG/HP‐treated cells, when compared to control cells, a phenomenon reversed by Decr1 knockdown (Figure 7a). The interaction between HADHA and HDAC3 was elevated under HG/HP conditions, but diminished following Decr1 knockdown (Figure 7b). Notably, HG/HP treatment promoted HDAC3 phosphorylation and mitochondrial translocation, a process prevented by Decr1 siRNA (Figure 7c–f). Overall, we unveiled that Decr1 upregulated the expression and kinase activity of PDK4, which in turn promoted HDAC3 phosphorylation and mitochondrial translocation. HDAC3 induced deacetylation of HADHA that exacerbated mitochondrial FAO and damage, leading to the onset and development of DCM.

### 3.8 Atranorin and Kurarinon Prevented DCM in T2D Mice by Binding to and Inhibiting Decr1

**FIGURE 8:**
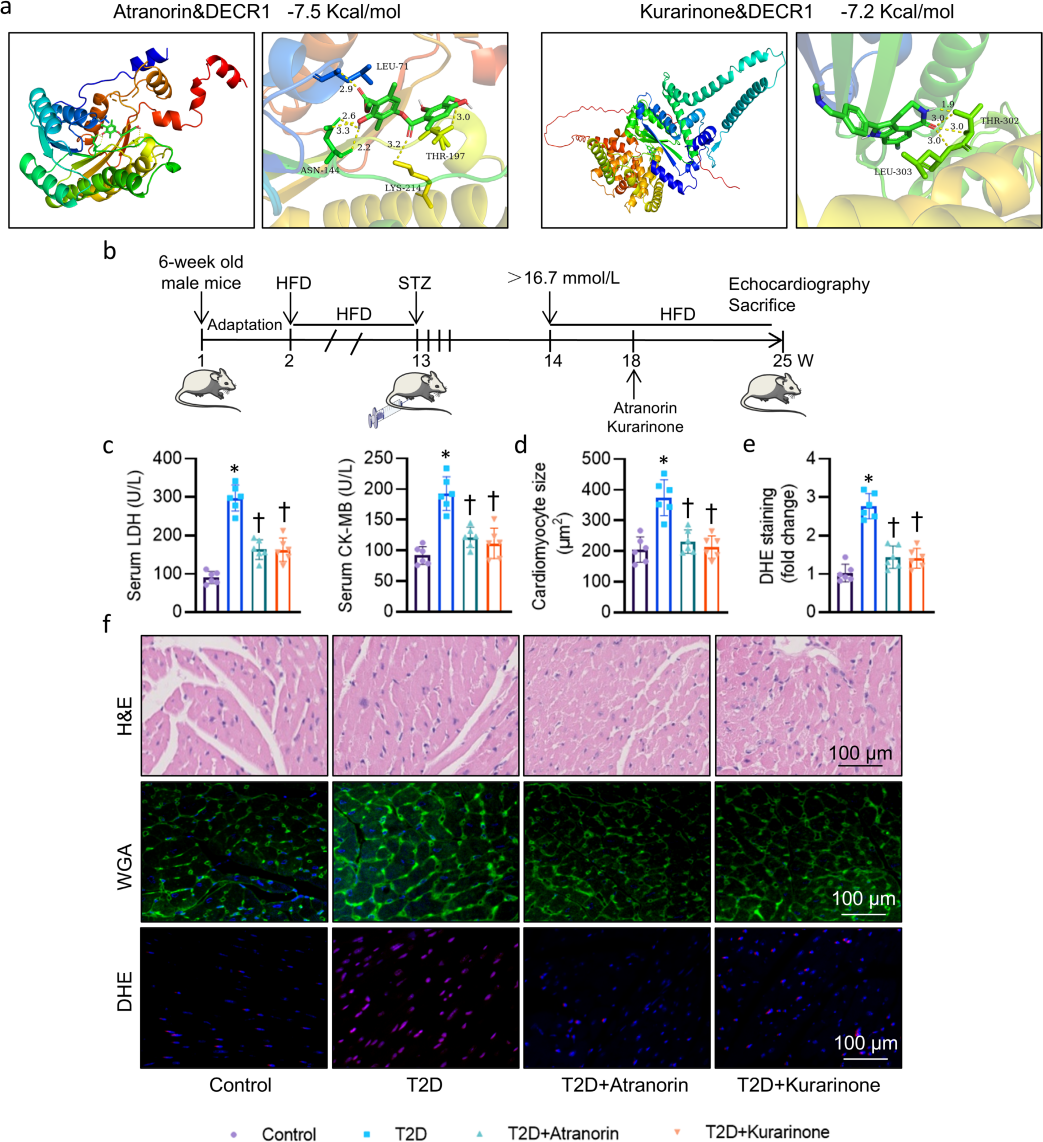
Atranorin and Kurarinon prevented DCM in T2D mice by binding to and inhibiting Decr1. (a) Molecular docking showing the direct binding of Atranorin to Decr1, and Kurarinon to Decr1. (b) The flow chart of animal experiments. (c) Serum LDH level and CK‐MB level. (d) Cardiomyocyte size measured by WGA staining. At least 100 cells measured in different visual fields from six samples per group. Each point on each column represents the average area of 100 myocardial cells in each sample. The quantification of average cross‐sectional area of cardiomyocytes compared with control in the indicated groups was shown in the bar graph. (e) The quantification of DHE staining. (f) Representative images of H&E staining, WGA staining, and DHE staining of left ventricle myocardium. Data were calculated as means ± SD. *p < 0.05 vs. Control. †*p* < 0.05 vs. T2D.

Because of the importance of Decr1 in DCM, we sought to determine whether pharmacologic inhibition of Decr1 may be useful for the management of DCM. To identify the potential inhibitors of Decr1, we established the cardiomyocytes expressing a luciferase reporter driven by a Decr1‐containing promoter. From a small molecule pool containing 256 natural products, both Atranorin and Kurarinon were found to decrease the Decr1‐luciferase activity by more than 70% (Figure S14a–c). Molecular docking revealed a direct interaction between Atranorin/Kurarinon and Decr1 with binding energy of −7.5 KJ/mol and −7.2 KJ/mol, respectively (Figure 8a). In support, DARTS also confirmed that Atranorin and Kurarinon directly bound to Decr1 (Figure S14d–e). Atranorin and Kurarinon treatment had negligible cytotoxicity in cultured cardiomyocytes, even at the higher concentrations (Figure S15a–b), and inhibited Decr1 protein and mRNA expression levels in HG/HP‐incubated cardiomyocytes (Figure S15c–e). Importantly, Atranorin and Kurarinon treatment increased cardiomyocyte resistance to HG/HP‐induced hypertrophy gene markers (Figure S15f). Afterwards, we assessed the pharmacological potential of Atranorin and Kurarinon in DCM mice (Figure 8b, S16a). Atranorin and Kurarinon treatment did not affect hyperglycemia, hyperlipidemia and body weight induced by STZ and HFD (Table S8). In spite of this, Atranorin and Kurarinon significantly prevented T2D‐increased serum LDH and CK‐MB levels by inhibiting the protein expression of Decr1 (Figure 8c). Echocardiography data showed that Atranorin and Kurarinon treatment obviously improved the impaired heart function in T2D mice (Figure S16b). H&E and WGA staining showed that the structural abnormalities and increased cardiomyocyte size were completely abrogated in mice treated with Atranorin and Kurarinon (Figure 8d,f). Sirius red staining showed that increased collagen deposition in diabetic heart was also prevented by Atranorin and Kurarinon (Figure S16c–d). Administration of Atranorin and Kurarinon attenuated excessive apoptosis and oxidative damage in diabetic heart, as reflected by TUNEL analysis (Figure S16c,e) and DHE staining (Figure 8e,f). In accordance with cardiac structures, supplementation of Atranorin and Kurarinon hindered the gene markers of cardiac hypertrophy and fibrosis (Figure S17). These data validated that Atranorin and Kurarinon might serve as promising inhibitors of Decr1, showing the therapeutic potential for DCM.

## 4 Discussion

In this study, we found higher expression of Decr1 in DCM through bioinformatics analysis sourced from public GEO databases, and this was replicated in our established DCM mice and HG/HP‐exposed cardiomyocytes. Functional gain and loss experiments showed that cardiomyocytes‐specific knockdown of Decr1 reduced cardiac pathological changes of DCM mice, while Decr1 overexpression exacerbated cardiac dysfunction and injury. Similar results were re‐produced in primary cardiomyocytes. Mechanistically, interaction of Decr1 with PDK4 induced PDK4 upregulation, leading to HDAC3 phosphorylation and mitochondrial translocation. In the mitochondria, HDAC3 facilitated HADHA deacetylation and induced aberrant mitochondrial FAO and injury, contributing to cardiac insult in diabetes. By binding to and inhibiting Decr1 expression, Atranorin and Kurarinon showed an ameliorative effect on DCM. This study highlights the essential role of Decr1 in cardiac abnormalities in the context of diabetes.

Bioinformatics analysis has shown that Decr1 may be dysregulated in patients with postischemic heart failure [16]. Weighted gene co‐expression network analysis has revealed that Decr1 was significantly increased in diabetic heart [17, 18]. The proteomic results showed that Decr1 protein may be a potential diagnostic and therapeutic target for obesity‐induced cardiovascular disease [19]. Decr1‐mediated mitochondrial lipid oxidation was observed in diabetic heart [20]. Nonetheless, no experiments were conducted to investigate the role and mechanisms of Decr1 in DCM. In this study, we investigated the role of Decr1 in DCM using both genomic analysis and experimental models. Through a comprehensive analysis of publicly available genomic data from the GEO database, we identified several genes with consistent expression changes in DCM, including Decr1. Specifically, Decr1 mRNA levels were significantly elevated in the hearts of diabetic mice, as well as in cardiomyocytes exposed to HG/HP, indicating a potential role in the pathogenesis of DCM. Further analysis using the HUMAN PROTEIN ATLAS and single‐cell sequencing confirmed that Decr1 is highly expressed in cardiomyocytes. Regarding DECR1 expression in cardiomyocyte clusters, we observed elevated DECR1 levels in clusters 0 and 1 from HUMAN PROTEIN ATLAS. Since DECR1 is involved in mitochondrial FAO, suggesting that these clusters may indeed have differences in FAO activity. Although we have not specifically measured mitochondrial FAO across these clusters, DECR1 expression may reflect varying metabolic requirements or oxidative stress responses within cardiomyocyte subsets. It is deserved to consider additional experiments to further explore FAO activity differences among different clusters in future studies. To explore the functional significance of Decr1 in DCM, we performed cardiac‐specific knockdown and overexpression experiments in diabetic mice. Cardiac‐specific knockdown of Decr1 significantly ameliorated diabetes‐induced cardiac dysfunction, as evidenced by improved left ventricular EF and FS. Additionally, Decr1 deficiency reduced cardiac hypertrophy, fibrosis, apoptosis, and oxidative stress in T2D mice. Conversely, cardiac‐specific overexpression of Decr1 exacerbated diabetes‐induced cardiac remodelling and dysfunction. In summary, our study demonstrates that Decr1 serves as a potential therapeutic target for mitigating diabetes‐related cardiac complications. Further studies are warranted to explore its potential as a therapeutic intervention in DCM in clinical practice. Moreover, construction of cardiomyocytes‐specific knockout of Decr1 may further confirm the importance of Decr1 in DCM‐induced cardiac dysfunction.

Mitochondria are the primary site of cellular energy production through OXPHOS in the electron transport chain (ETC). During this process, electrons are transferred through complexes I‐IV of the ETC, which ultimately leads to the formation of ATP. However, some electrons can ‘leak’ from complexes I and III, prematurely reacting with oxygen to form superoxide, a type of ROS. This is a natural byproduct of mitochondrial respiration. Excessive ROS production, often due to mitochondrial dysfunction or high metabolic activity, can overwhelm the cell's antioxidant defences. This leads to oxidative stress, causing damage to mitochondrial DNA, proteins, and lipids, which can further impair mitochondrial function. This dysfunctional cycle can exacerbate ROS production, leading to cellular damage in DCM. In this study, we found that Decr1 disrupted the activities of mitochondrial complexes I, II, III and IV, thereby reducing cardiac ATP contents in the settings of DCM. These results suggested that Decr1 contributes to DCM development by inducing mitochondrial damage and ROS overproduction.

Our study revealed a novel mechanistic pathway in DCM involving the upregulation of Decr1 and its downstream effects on PDK4‐mediated metabolic dysregulation in cardiomyocytes. RNA sequencing analysis demonstrated that Decr1 overexpression in cardiomyocytes led to the upregulation of 235 genes and the downregulation of 574 genes, with PDK4 emerging as a prominently upregulated target. This was confirmed by RT‐PCR and Western blotting, which showed significantly elevated PDK4 expression in Decr1‐overexpressed cardiomyocytes, particularly under HG/HP conditions. Notably, Decr1 silencing reduced PDK4 expression, further suggesting that Decr1 is an upstream regulator of PDK4 in the context of DCM. Functionally, PDK4 downregulation attenuated HG/HP‐induced hypertrophy, apoptosis, and oxidative damage in cardiomyocytes, highlighting the pathogenic role of Decr1 as a key mediator of diabetes‐induced cardiac injury. Furthermore, we demonstrated that PDK4 overexpression abolished the protective effects of Decr1 deficiency in diabetic mice, underscoring the critical interplay between Decr1 and PDK4 in driving DCM pathology. In addition, our data indicate that Decr1 interacted directly with PDK4, as evidenced by immunoprecipitation, GST pulldown assays, and confocal microscopy. This interaction appears to enhance PDK4 activity, leading to increased FAO and decreased glucose oxidation, a metabolic shift that is detrimental in the diabetic heart. Indeed, Decr1 deficiency restored the impaired glucose and pyruvate oxidation rates observed in diabetic cardiomyocytes, suggesting that targeting Decr1 could normalize the metabolic disturbances characteristic of DCM. Notably, transcriptome analysis showed that Decr1 might regulate a host of gene levels in DCM, not only PDK4. Examining additional mRNA targets of Decr1 would provide further insights and could reveal complementary or synergistic metabolic pathways affected in DCM. Future studies are required to explore these other mRNAs to understand their potential roles and interactions in regulating metabolic adaptation. In addition, 10 GO items significantly enriched were identified, including fatty acid metabolism process, lipid metabolic process, xenobiotic metabolic process, to name a few. Future studies are warranted to examine whether Decr1 had a role in DCM by regulating other metabolic processes.

HDAC3 is a key enzyme involved in acetylation, and HDAC3 can translocate into the mitochondria and induce the deacetylation of the mitochondrial fatty acid β‐oxidation enzyme HADHA [13]. This disruption of mitochondrial homeostasis results in excessive oxidative stress, which is thought to contribute to cardiac injury. Additionally, we discovered that PDK4 facilitated the phosphorylation and mitochondrial translocation of HDAC3, which subsequently deacetylated HADHA, a critical enzyme in mitochondrial FAO. The deacetylation of HADHA, driven by HDAC3, exacerbates mitochondrial dysfunction, contributing to the metabolic and structural derangements seen in DCM. In summary, our findings identify Decr1 as a pivotal regulator of PDK4 in the diabetic heart, where it drives a cascade of events leading to enhanced FAO, reduced glucose oxidation, and mitochondrial dysfunction. These results not only expand our understanding of the molecular mechanisms underlying DCM but also suggest that targeting the Decr1‐PDK4 axis could offer a therapeutic strategy to ameliorate DCM.

Given the critical role of Decr1 in the pathological processes of DCM, the identification of potential inhibitors is crucial for developing effective treatments. Through a screening of our phytochemical compound library using a luciferase reporter assay for Decr1 activity, we identified the phytochemical Atranorin and Kurarinone as direct inhibitors of Decr1, effectively inhibiting its expression. Both cellular and animal studies demonstrated that Atranorin and Kurarinone treatment significantly improved DCM pathologies. These findings suggest that Atranorin and Kurarinone are promising candidates for DCM therapy. While Atranorin and Kurarinone were identified as inhibitors of Decr1, the specificity of these compounds for Decr1 over other related enzymes or pathways has not been fully established. Off‐target effects could contribute to the observed improvements in DCM pathology, potentially confounding the results. While the current animal studies demonstrate a therapeutic effect, the long‐term efficacy and safety of Atranorin and Kurarinone need to be evaluated in more extensive in vivo studies. Potential side effects, toxicity, and pharmacokinetic profiles must be thoroughly investigated before these compounds can be considered viable therapeutic options. Given the role of Decr1 in fatty acid metabolism, future studies could explore the potential benefits of Decr1 inhibitors in other cardiometabolic diseases, such as obesity‐related cardiomyopathy or heart failure with preserved ejection fraction (HFpEF).

This study suggests that inhibiting Decr1 might hold significant potential for managing DCM. Molecular mechanism studies showed that Decr1 upregulated PDK4 expression and kinase activity, leading to enhanced HDAC3 phosphorylation and mitochondrial translocation. Phosphorylated HDAC3 induces HADHA deacetylation, exacerbating mitochondrial fatty acid oxidation and contributing to mitochondrial damage, ultimately leading to DCM. These findings not only position Decr1 at the core of the cellular processes contributing to DCM pathogenesis but also hint at its utility in developing complementary treatment strategies. One should bear in mind that the myocardium undergoes various forms of metabolic pathways, including FAO, glucose oxidation, lactate oxidation, ketone body oxidation, amino acid metabolism, oxidative phosphorylation, phosphocreatine shuttle, pentose phosphate pathway. Our study specifically examined the regulation of FAO by Decr1 using RNA sequencing and functional experiments, without addressing other forms of metabolic pathways. Future research will explore whether Decr1 also influences other cardiac metabolic pathways. Additionally, investigating cardiac‐specific knockout models of Decr1 will provide further insights into its full range of biological activities. In addition, DECR1 is primarily localized within the mitochondria, where it plays a key role in mitochondrial fatty acid β‐oxidation, particularly in the breakdown of unsaturated fatty acids. Its mitochondrial localization aligns with its function in lipid metabolism, as DECR1 is essential for reducing intermediates in the FAO pathway to facilitate energy production. In this study, we did not perform specific localization assays to confirm the subcellular distribution of DECR1. However, based on established literature and the functional role of DECR1, we expect it to be predominantly mitochondrial. However, it cannot be ruled out that Decr1 may exert its effects in physiological or pathological states by leaking out of mitochondria into the cytoplasm or other organelles. Future studies are highly recommended to use immunofluorescence or fractionation techniques to validate and visualize its precise localization within cardiomyocytes and other cell types in health and diseases.

## Conflicts of Interest

The authors declare no conflicts of interest.

## Supporting information


**Data S1** Supplementary Information.

**Fig. S1** The expression paradigm of Decr1. (a) Decr1 is expressed in all organs throughout the body, with its expression being most pronounced in the cardiomyocytes in the HUMAN PROTEIN ATLAS database. (b) Decr1 expression levels in different cells of human heart. (c) Decr1 displayed the highest expression in the cardiomyocytes of the heart. (d) The immunohistochemical staining of Decr1 in the heart tissues from the HUMAN PROTEIN ATLAS database. (e) Decr1 was mainly expressed in cardiomyocytes using the Tabula Muris database.
**Figure S2.** Decr1 expression is elevated in the heart of T2DM mice and HG/HP‐exposed cardiomyocytes. (a) The protein expression of Decr1 in the heart of control and T2D mice. (b) Immunohistochemistry of Decr1 in the heart. (c) Immunofluorescence showing the co‐localization of Decr1 and cardiomyocyte marker. (d) Relative mRNA level of Decr1 in isolated cardiomyocytes, cardiac fibroblasts, and cardiac endothelial cells. (e) The protein expression of Decr1 in HG/HP‐exposed cardiomyocytes. (f) Relative mRNA level of Decr1 in HG/HP‐exposed cardiomyocytes. Data were calculated as means ± SD. *p < 0.05 vs. Control (Con) or normal glucose (NG).
**Figure S3.** Cardiac‐specific deficiency of Decr1 alleviates DCM in T2D mice. (a) The protein expression of Decr1. (b) EF and FS. (c) Representative images of Sirius Red staining and TUNEL staining. (d) The quantification of cardiac fibrosis measured by Sirius Red staining. (e) The quantitative analysis of TUNEL staining. (f) The activity of Complex I, Complex II, Complex III, and Complex IV. (g) ATP contents. Data were calculated as means ± SD. *p < 0.05 vs. ShCon. †*p* < 0.05 vs. T2D + ShCon.
**Figure S4.** Cardiac‐specific overexpression of Decr1 aggravates DCM in T2D mice. (a) The flow chart of animal experiments. (b) The protein expression of Decr1. (c) Serum LDH level and CK‐MB level. (d) EF and FS. (e) Representative images of HE staining, WGA staining, Sirius Red staining, TUNEL staining and DHE staining. (f) Cardiomyocyte size analysed by WGA staining of left ventricle myocardium. At least 100 cells measured in different visual fields from six samples per group. Each point on each column represents the average area of 100 myocardial cells in each sample. The quantification of average cross‐sectional area of cardiomyocytes compared with control in the indicated groups was shown in the bar graph. (g) The quantitative analysis of cardiac fibrosis. (h) The quantitative analysis of TUNEL staining. (i) The quantitative analysis of DHE staining. (j) Relative mRNA level of *Nppa*, *Nppb*, *Myh7*, *Col1a1*, and *Tgfb1*. Data were calculated as means ± SD. *p < 0.05 vs. Vector. †*p* < 0.05 vs. T2D.
**Figure S5.** Cardiac‐specific overexpression of Decr1 aggravates mitochondrial damage in the heart of DCM mice. (a) The activity of Complex I, Complex II, Complex III, and Complex IV. (b) ATP contents. Data were calculated as means ± SD. *p < 0.05 vs. Vector. †*p* < 0.05 vs. T2D.
**Figure S6.** The protein and mRNA expression levels of Decr1 in cardiomyocytes in vitro. (a, b) The protein expression of Decr1 in cardiomyocytes after Decr1 knockdown. (c) The mRNA level of Decr1 in cardiomyocytes after Decr1 knockdown. (d, e) The protein expression of Decr1 in cardiomyocytes after Decr1 overexpression. (f) The mRNA level of Decr1 in cardiomyocytes after Decr1 overexpression. Data were calculated as means ± SD. *p < 0.05 vs. Con siRNA or Vector.
**Figure S7.** Downregulation of Decr1 inhibited HG/HP‐induced injury in cardiomyocytes in vitro*.* (a) LDH release. Data were calculated as means ± SD. *p < 0.05 vs. Con siRNA. †*p* < 0.05 vs. HG/HP + Con siRNA.
**Figure S8.** Upregulation of Decr1 worsens HG/HP‐induced injury in cardiomyocytes. (a) LDH release. (b) Relative mRNA level of *Nppa*, *Nppb, Myh7*. (c) Cardiomyocyte size measured by α‐actinin staining. (d) Representative images of α‐actinin staining, TUNEL staining, DHE staining, mitochondrial ROS levels, and JC‐1 staining. (e) Quantification of TUNEL staining. (f) Quantification of DHE staining. (g) Quantification of mitochondrial ROS levels. Data were calculated as means ± SD. *p < 0.05 vs. Vector. †*p* < 0.05 vs. HG/HP.
**Figure S9.** Effects of Decr1 on the protein expression of PDK4 in cardiomyocytes. (a–c) The protein expression of PDK4 and Decr1 in cardiomyocytes after overexpression of Decr1. (d–f) The protein expression of PDK4 and Decr1 in cardiomyocytes after knockdown of Decr1. Data were calculated as means ± SD. *p < 0.05 vs. pcDNA3.1 or Con siRNA. †*p* < 0.05 vs. HG/HP + pcDNA3.1 or HG/HP + Con siRNA.
Figure S10. The protein expression of PDK4 after transfection of PDK4 siRNA. Data were calculated as means ± SD. *p < 0.05 vs. Con siRNA.
**Figure S11.** Overexpression of PDK4 blocked the benefits of Decr1 knockdown in DCM. (a) EF and FS. (b) Sirius Red staining, and TUNEL staining of left ventricle myocardium. (c) Representative images of Sirius Red staining and TUNEL staining. (d) The quantification of cardiac fibrosis measured by Sirius Red staining. (e) The quantitative analysis of TUNEL staining. Data were calculated as means ± SD. *p < 0.05 vs. T2D. †*p* < 0.05 vs. T2D + ShDecr.
**Figure S12.** The direct interaction of Decr1 and PDK4. (a) The co‐IP assays in HEK293 cells transfected with Flag‐tagged PDK4 and HA‐tagged Decr1. Anti‐Flag and anti‐HA antibodies were used as western blot probes. (b) The co‐IP assays in HEK293 cells transfected with Flag‐tagged Decr1 and HA‐tagged PDK4. Anti‐Flag and anti‐HA antibodies were used as western blot probes. (c) GST precipitation assays showing direct PDK4‐Decr1 binding. Purified GST was used as a control. (d) Results from co‐IP assays showing the binding regions between Decr1 and PDK4. (e) The co‐localization of PDK4 and Decr1 assessed by laser confocal microscope. (f) The interaction of PDK4 with Decr1 in cardiomyocytes upon exposure of HG/HP. Data were calculated as means ± SD. *p < 0.05 vs. NG.
**Figure S13.** Suppression of Decr1 P‐PDH expression and restored glucose oxidation in diabetic cardiomyocytes. (a) The phosphorylation level of PDH. (b) Kinetic oxygen consumption rate (OCR) responses of isolated cardiomyocytes to 10 mM glucose. (c) Calculated glucose oxidation rate. (d) Kinetic OCR responses of isolated cardiomyocytes to 1 mM pyruvate. (e) Calculated pyruvate oxidation rate. The glucose or pyruvate oxidation rate was calculated by the OCR increase. Data were calculated as means ± SD. *p < 0.05 vs. Con siRNA or NG. †*p* < 0.05 vs. HG/HP + Con siRNA or HG/HP.
**Figure S14.** Atranorin and Kurarinon show strong ability to inhibit Decr1 activity. (a) Heatmap showing the effects of different compounds on the activity of Decr1. (b) Top 10 compounds showed the suppressive effects on the activity of Decr1. (c) Chemical structure of Atranorin and Kurarinon. (d) DARTS showing the direct binding of Atranorin to Decr1 protein. (e) DARTS showing the direct binding of Kurarinon to Decr1 protein.
**Figure S15.** Atranorin and Kurarinon protect cardiomyocytes against HG/HP‐induced injury. (a) Cell viability after treatment with Atranorin. (b) Cell viability after treatment with Kurarinon. (c, d) The protein expression of Decr1. (e) The mRNA level of Decr1. (f) Relative mRNA level of *Nppa*, *Nppb*, *Myh7*. Data were calculated as means ± SD. *p < 0.05 vs. Control. †*p* < 0.05 vs. HG/HP.
**Figure S16.** Atranorin and Kurarinon improved DCM by binding to and suppressing Decr1. (a) The protein expression of Decr1. (b) EF and FS. (c) Representative images of Sirius Red staining and TUNEL staining. (d) The quantification of cardiac fibrosis measured by Sirius Red staining. (e) The quantitative analysis of TUNEL staining. Data were calculated as means ± SD. *p < 0.05 vs. Control. †*p* < 0.05 vs. T2D.
**Figure S17.** Atranorin and Kurarinon prevented cardiac hypertrophy and fibrosis in T2D mice. Relative mRNA level of *Nppa*, *Nppb*, *Myh7*, *Col1a1*, and *Tgfb1*. Data were calculated as means ± SD. *p < 0.05 vs. Control. †*p* < 0.05 vs. T2D.
Table S1: Table S1. Information for primary and secondary antibodies.
Table S2. Primers for Real‐time quantitative PCR analysis in mice.
Table S3. Primers for Real‐time quantitative PCR analysis in cells.
Table S4. Compound information used for screening experiments.
Table S5. The body weight, biochemical characteristics and echocardiographic data in each group.
Table S6. The body weight, biochemical characteristics and echocardiographic data in each group.
Table S7. The body weight, biochemical characteristics and echocardiographic data in each group.
Table S8. The body weight, biochemical characteristics and echocardiographic data in each group.

## Data Availability

Source data for all main figures and extended data figures are supplied with this paper. Experimental data supporting the plots within this paper and other findings of this study are available from the corresponding author upon reasonable request.
