# Supplementary material for: Therapeutic Targeting of Decr1 Ameliorates Cardiomyopathy by Suppressing Mitochondrial Fatty Acid Oxidation in Diabetic Mice
Source: J Cachexia Sarcopenia Muscle. 2025 Mar 7;16(2):e13761. doi: 10.1002/jcsm.13761 (PMC11886612; doi:10.1002/jcsm.13761)
Supplement: Supplementary file 1 — Data S1 Supplementary Information. [file JCSM-16-e13761-s001.docx]

**1.1 Reagents and chemicals**

Dulbecco's Modified Eagle's Medium (DMEM), trypsin-EDTA, and fetal bovine serum (FBS), were sourced from Hyclone (South Logan, UT, USA). RIPA lysis buffer was obtained from ThermoFisher (Waltham, MA, USA), and cell culture plates from Corning (Corning, CA, USA). Hematoxylin &Eosin (HE) staining kits (G1120) and Sirius Red staining kits (G1472) were purchased from Solarbio LIFE SCIENCES (Beijing, China). Click-iT™ Plus TUNEL Assay for in situ apoptosis detection, and Alexa Fluor™ 488 dye, Click-iT™ Plus TUNEL Assay for in situ apoptosis detection, and Alexa Fluor™ 594 dye, MitoSOX™, Lipofectamine 3000 and wheat germ agglutinin (WGA) were procured from Invitrogen (Carlsbad, CA, USA). Lactate dehydrogenase (LDH) detection kit, mito-tracker green probe, cell mitochondrial isolation kit, Cell Counting Kit-8 (CCK8), and mitochondrial membrane potential detection kit (JC-1) were obtained from Beyotime (Shanghai, China). Mouse Creatine Kinase MB (CK-MB) ELISA Kit was supplied by Abcam (Cambridge, MA, USA). Dihydroethidium (DHE), Streptozocin (STZ), palmitic acid (PG), glucose, pyruvate, streptozocin (STZ), DAPI, and Pronase E were supplied by Sigma-Aldrich (St. Louis, MO, USA). The information for primary and secondary antibodies was provided in supplementary **Table S1**.

**1.2 Animals**

All animal experimental procedures were conducted in accordance with the Guide for the Care and Use of Laboratory Animals (8th edition, revised 2011), as outlined by the US National Institutes of Health. Approval for the study was granted by the Laboratory Animal Welfare & Ethics Committee of Jiangnan University. Six-week old male C57BL/6J mice were used throughout the experiment (Gempharmatech Co., Ltd., Nanjing, China). The mice were housed in the laboratory animal center at Jiangnan University, maintained under constant temperature and humidity, with unrestricted access to food and water. They were kept under a controlled 12-h light/dark cycle. To induce a type 2 diabetes (T2D) model, mice were fed a high-fat diet (HFD, 60% fat of total kcal) and subjected to intraperitoneal injections of streptozotocin (STZ), according to previously established protocols for T2D models ^1, 2^. After 11 weeks on HFD, mice received three consecutive daily intraperitoneal injections of STZ (40 mg/kg) dissolved in 0.1 M pre-cooled citrate buffer (pH 4.5), prepared immediately prior to use. Control mice were injected with citrate buffer only. Mice with fasting blood glucose levels above 16.7 mmol/L, measured from tail vein blood, were diagnosed as diabetic. These mice continued on the HFD until sacrifice at 31 weeks of age. Adeno-associated virus serotype 9 (AAV9) vectors containing the cardiomyocyte-specific cTnT promoter were designed and synthesized. To specifically knock down Decr1 in the heart, AAV9 vectors carrying Decr1 shRNA or non-target shRNA, also under the cTnT promoter, were constructed. The sequences for Decr1 shRNA were 5'-CCGGCCCGACTGGAAGATTTGAGAACTCGAGTTCTCAAATCTTCCAGTCGGGTTTTTG-3', while the control shRNA sequences were 5′-TTCTCCGAACGTGTCACGT-3′. The full-length Decr1 gene was cloned into the AAV9 plasmid to achieve cardiac-specific Decr1 overexpression (OE) under the cTnT promoter, with blank AAV9 vectors serving as negative controls. To specifically overexpress Decr1 in murine hearts, mice were injected with AAV9 vectors carrying Decr1 under the control of a cTnT promoter, or with negative control AAV9 (1 × 10¹¹ viral genome particles per mouse), administered four weeks after the initial STZ injection. Similarly, for Decr1 knockdown, mice received tail vein injections of AAV9 vectors carrying Decr1 shRNA or control shRNA (1 × 10^11^ viral particles per mouse), also under the control of the cTnT promoter, four weeks after the first STZ injection. To investigate the effects of Atranorin and Kurarinone on DCM, mice were administered intraperitoneal injections of Atranorin (10 mg/kg) and Kurarinone (10 mg/kg) every other day for 8 weeks. FBG levels were measured and recorded at the end of the experiment. At the end of the present study, mice were anesthetized with 1%–2% isoflurane and euthanized by cervical dislocation.

**1.3 Cell culture**

Neonatal rat cardiomyocytes were isolated from 1–3 day-old Sprague-Dawley rats, and cardiac endothelial cells and fibroblasts from mice were cultured as we previously described ^3, 4^. The mRNA level of Decr1 was measured in cardiac fibroblasts and endothelial cells from both control and diabetic mice. Neonatal rat cardiomyocytes were seeded in six-well plates at a density of 2.5 × 10⁴ cells/well and cultured in low-glucose (1 g/L) DMEM supplemented with 10% FBS, gentamicin (50 μg/ml), and cytosine β-D-arabinofuranoside (Ara-C, 10 μM) to inhibit cardiac fibroblast growth. After 12 h of synchronization, cells were treated with high glucose (HG, 33.3 mM) or and palmitic acid (HP, 100 μM) for 72 h to simulate diabetic cardiac injury *in vitro*. For Decr1 knockdown, primary cardiomyocytes were transfected with scramble shRNA (TR30013, 0.5 μg, OriGene) or Decr1 shRNA (TR712397, 0.5 μg, OriGene) using Lipo3000™ transfection reagents (RiboBio, China). Neonatal cardiomyocytes were also transfected with lentivirus vectors for Decr1 overexpression (RR208399L3, 1 × 10^6^ TU/mL, OriGene) or control vectors (PS100092V, 1 × 10^6^ TU/mL, OriGene) for 24 h, followed by 72 h of HG/HP treatment. After that, cardiomyocyte hypertrophy, apoptosis, and oxidative stress were then measured.

**1.4 Serum biochemical parameters**

The blood samples and heart tissues were collected. Blood was centrifuged at 3000 rpm for 15 min and serum was collected and stored at -20 °C for the determination of serum biochemical parameters. Serum levels of creatine kinase-MB (CK-MB) and lactate dehydrogenase (LDH) were measured using commercial kits purchased from ERBA Diagnostics (Germany), following the manufacturer's instructions with a microplate reader. Biochemical indicators, including fasting blood glucose (FBG), low density lipoprotein (LDL), total cholesterol (TC) and triglycerides (TG) were measured.

**1.5 Real time-polymerase chain reaction (RT-PCR)**

RNA extraction using TRIzol was performed, followed by phase separation with chloroform, RNA precipitation with isopropanol, washing with ethanol, and final suspension in RNase-free water. cDNA synthesis was carried out using the SuperScript™ III First-Strand Synthesis SuperMix kit. Gene amplification and expression analysis were performed on the Real-Time System (Rocher) with SYBR Green detection and specific primers (**Supplementary Table S2-3**). β–actin was used as the reference gene for normalization.

**1.6 Western blot and co-immunoprecipitation**

For western blot analysis, cardiac tissues and cell samples were homogenized in radioimmunoprecipitation assay (RIPA) buffer using a Sonic Dismembrator. Protein concentrations were determined using the Bio-Rad Protein Assay Solution (Bio-Rad) and measured with a microplate reader. A total of 30 µg of protein was loaded onto Bolt™ Bis-Tris Mini Protein Gels (ThermoFisher) and transferred to polyvinylidene fluoride (PVDF) membranes. Membranes were blocked with 5% BSA or nonfat dry milk in Tris-buffered saline with Tween-20, followed by overnight incubation at 4°C with the following primary antibodie. The membranes were then incubated with horseradish peroxidase (HRP)-conjugated secondary antibody for 1 h at room temperature. Protein bands were visualized using Pico PLUS Chemiluminescent Substrate (ThermoFisher). ImageJ software was used to analyze the greyscale values. The ratio of the targeted protein band density to the β–actin protein band density was used for statistical analysis. For co-immunoprecipitation assays, sample were lysed in RIPA buffer and the lysates were incubated overnight at 4°C with protein A/G agarose beads (Santa Cruz, CA, USA) and 1 μg of the indicated antibodies or control IgG. The beads were then washed three times with cold IP buffer, and the immunoprecipitates were analyzed by Western blot.

**1.7 Immunohistochemistry**

During the immunohistochemistry procedure, cardiac sections (5 μm) were treated with a 10% H_2_O_2_ solution for 30 min to block endogenous peroxidase activity. The sections were then blocked with 10% normal goat serum for 1 h at room temperature. Following this, sections were incubated with primary antibodies against Decr1 at 4°C for 24 h. Antibody detection was performed using the Histostain-Plus Bulk kit (Bioss, Inc.), with visualization by 3,3′-diaminobenzidine (DAB). The sections were rinsed in phosphate-buffered saline (PBS) and imaged using an Olympus C-5050 digital camera mounted on an Olympus BX51 microscope. Positive immunoexpression was indicated by brown cytoplasmic staining. Quantification of positive cells was performed by assessing at least fifty cardiac muscle cells per field in six different fields of each tissue section.

**1.8 Immunofluorescence staining**

To investigate the localization of Decr1 in cardiomyocytes, immunofluorescence double staining was performed using antibodies against Decr1 and sarcomeric actin. After sacrifice, cardiac sections were permeabilized with 0.1% Triton X-100 for 15 minutes and incubated with rabbit anti-Decr1 and mouse anti-sarcomeric actin antibodies. Sections were then treated with Alexa Fluor® 594-conjugated Goat anti-Rabbit IgG and Alexa Fluor® 488-conjugated Goat anti-Mouse IgG for 1 h. The collected cardiomyocytes were fixed with 4% paraformaldehyde for 30 min, blocked with 5% BSA for 1 h, and incubated with anti-α-actinin antibody at 4°C overnight. This was followed by a 1-h incubation with Alexa Fluor® 594-conjugated Goat anti-Mouse IgG H&L. Immunofluorescence signals were visualized using a Nikon 80i fluorescence microscope (Tokyo, Japan).

**1.9 Echocardiography**

Mice were shaved to remove chest hair and fully exposed at the forehead. Anesthesia was induced via isoflurane inhalation, and the mice were positioned on a thermostatically heated plate with limbs extended. Ultrasound gel was applied to the chest, and left ventricular short-axis views were obtained using echocardiography system equipped with 30 MHz probe (Visualsonics, Toronto, Canada). Two-dimensional images were captured, and left ventricular ejection fraction (LVEF), left ventricular shortening fraction (LVFS), and left ventricular end-diastolic volume (LVEDV) were calculated using M-mode. Each mouse underwent at least six measurements, and the average of three cardiac cycles was recorded.

**1.10 Histological examination**

Heart tissues were harvested, fixed, dehydrated, and embedded in paraffin. Myocardial sections (5 μm) were stained with hematoxylin and eosin (HE) for histopathology or Sirius Red to assess collagen deposition using an Olympus BX50 microscope. FITC-conjugated wheat germ agglutinin (WGA, Invitrogen Corp) was used to evaluate myocyte cross-sectional area, and DAPI was applied to identify nuclei. Myocyte size was measured using Image Pro-Plus version 6.0, with a minimum of 100 myocytes analyzed per mouse in each group. Collagen and non-collagen components appeared as red and orange stains, respectively. The fibrotic area was calculated as the ratio of myocardial collagen area to total field area using Image Pro-Plus version 6.0 (Media Cybernetics, Bethesda, MD, USA), with consistent parameters across samples. The percentage of stained tissue was determined in six fields per sample. Cardiac ROS levels measured using the fluorescent probe DHE as previously described ^5^. Cardiac sections were incubated with DHE (10 μM) for 30 minutes in a light-protected humidified chamber. After washing with PBS, fluorescence signals were visualized using a Leica DMi 8 fluorescence microscope (Leica Microsystems, Germany). Cardiac apoptosis was assessed using a commercial TUNEL assay kit, and the relative number of positive cells was quantified with Image J software.

**1.11 Mitochondrial ROS detection and JC-1 staining**

For mitochondrial ROS detection, cardiomyocytes were treated with MitoSOX Red (5 μM) for 30 min. Red fluorescence indicated mitochondrial superoxide, which was analyzed using the same fluorescence microscope. Mitochondrial ROS levels were quantified and normalized to control cells. Mitochondrial membrane potential (Δψm) was assessed using the fluorescent dye JC-1. Cells were incubated with JC-1 for 20 min at 37°C, followed by a PBS wash. Fluorescence images of JC monomers (green, 535 nm) and JC aggregates (red, 570 nm) were captured using a Leica DMi 8 fluorescence microscope (Leica Microsystems, Germany).

**1.12 RNA sequencing**

Transcriptome sequencing and analysis were performed by OE Biotech Co., Ltd. (Shanghai, China) as we previously described ^4^. The raw RNA sequencing data have been submitted to the Sequencing Read Archive (SRA) under accession number PRJNA1158945.

**1.13 Analysis of glycolysis, glucose oxidation, and fatty acid oxidation**

Isolated cardiomyocytes were seeded on matrix gel–coated Agilent Seahorse XF24 microplates at a density of 8000 cells for each well. Prior to the experiment, the culture medium was replaced with 500 μL of Agilent Seahorse XF Base Medium, and cells were incubated at 37°C in a non-CO_2_ incubator for 1 h. A sensor cartridge was loaded with 100 mM of glucose, 10 μM of oligomycin, and 1 M of 2-deoxy-glucose (2-DG) for calibration. The basal extracellular acidification rate (ECAR) was measured with the following protocol: 3 cycles of 1.5 min mixing, 2 min waiting, and 1.5 min measuring. This was followed by successive injections of glucose, oligomycin, and 2-DG, with ECAR measured after each injection using the same protocol. The sensor cartridge was loaded with 100 mM glucose or 10 mM pyruvate. Basal oxygen consumption rate (OCR) was measured using the same 3-cycle protocol (1.5 min mix, 2 min wait, 1.5 min measure), followed by fuel substrate–induced OCR measurements after glucose or pyruvate injection for measurement of glucose and pyruvate oxidation. Palmitate was conjugated with BSA for measurement of palmitate oxidation as previously described ^6, 7^. The sensor cartridge was loaded with 10 mM palmitate-BSA or BSA solution and injected after basal OCR measurement.

**1.14 Extraction of mitochondrial proteins**

The collected cells were gently suspended the cell pellet in pre-cooled PBS and centrifuged at 600g for 5 min at 4°C, and the supernatant was discarded. A total of 1 ml of mitochondrial isolation reagent was added and incubated in an ice bath for 15 min. The cell homogenate was centrifuged at 1000g for 10 min at 4°C. The supernatant was then carefully transferred to a new centrifuge tube and centrifuged at 11000g for 10 min at 4°C. The supernatant was non-mitochondrial proteins, and the precipitation was the separated cell mitochondria.

**1.15 Drug screening**

A small pool of 256 natural products was screened for their effects on Decr1 promoter activity using a luciferase reporter assay (**Table S4**). The full-length PGK1 promoter region (−2995 bp to the transcription start site) was cloned into the pGL3 luciferase vector (Promega) and transfected into cardiomyocytes for 48 h. These compounds (5 μM) were added in the presence of HG/HP for 24 h. Firefly luciferase activity was measured using a dual luciferase reporter assay kit (Promega).

**1.16 Molecular docking**

The structure of Decr1 (PDB ID: 7UCW) was obtained from the Protein Data Bank, and the structure of Atranorin or Kurarinone was retrieved from PubChem. Protein receptor molecules were prepared by AutoDockTools (version 4.2.6), with hydrogenation and charge calculation, using the binding site of the protein's ligand as the active pocket. Molecular docking was performed using Vina 1.5.6, and the 3D docking results were visualized with PyMOL software.

**1.17 Drug affinity responsive target stability (DARTS) assay**

Lysates from cardiomyocytes were diluted in 1× TNC buffer (Tris-HCl, NaCl, CaCl₂). Each aliquot was treated with DMSO or Atranorin or Kurarinone (5 μM) at room temperature for 1 h, followed by proteolysis with 0.05% pronase for 15 min. The reaction was stopped by adding 5× loading buffer (Solarbio, Beijing, China). Supernatants were analyzed by western blot to detect Decr1 protein expression.

**1.18 Laser confocal staining**

After washing three times with PBS, cardiomyocytes were fixed with 4% paraformaldehyde for 15 min. The cells were permeabilized with 0.5% Triton X-100 for 15 minutes, washed again with PBS, and blocked with 10% goat serum at room temperature for 30 min. Primary antibodies against Decr1 (1:200, rabbit polyclonal antibody, Biorybyt) and PDK4 (1:200, mouse monoclonal antibody, Abcam) were applied, and the cells were incubated overnight in the dark. On the following day, after reaching room temperature, the cells were washed twice with ice-cold 0.1% Tween and once with PBS. Secondary antibodies including Goat Anti-Mouse IgG H&L (Alexa Fluor® 488) (ab150113, 1:1000) and Goat Anti-Rabbit IgG H&L (Alexa Fluor® 594) (ab150080, 1:1000) were added, and incubation was carried out at room temperature in the dark for 1 h, followed by washing as described. Nuclei were stained with DAPI to ensure coverage. After three washes, the cells were observed under a laser confocal fluorescence microscope (LSM880 with Airyscan, Carl Zeiss, Germany).

**1.19 Plasmid constructs**

To determine the interaction of PDK4 with Decr1, sequencing encoding full-length of PDK4 with Decr1 were cloned into either pcDNA5-Flag or pcDNA5-hemagglutinin (HA) vectors to yield pcDNA5-Flag-PDK4, pcDNA5-HA-Decr1, pcDNA5-Flag-Decr1, pcDNA5-HA-Flag, respectively. Plasmids encoding pcDNA5-HA-GST-Decr1 and pcDNA5-HA-GST-PDK4 were acquired by cloning the indicated cDNA of Decr1 and PDK4 into pcDNA5-HA-GST vectors. The pcDNA-Flag-Decr1, psi-Flag-PDK4, and DNA constructs of Decr1 deletion fragments and PDK4 deletion fragments were attained by cloning the indicated cDNA of Decr1 and PDK4 into the pcDNA5-Flag and psi-Flag vectors, respectively. These plasmids were transfected to HEK293 cells, and their interactions were examined by immunoblotting.

**1.20 Measurement of Complex I, II, III, and IV activities**

Complex I, II, III, and IV activities were measured in frozen isolated mitochondrial samples from mouse hearts using a 96-well plate assay format ^8, 9^. Enzyme activities for Complexes I, II, and IV were assessed using the Complex I, II, and IV Enzyme Activity Microplate Assay Kits (MitoSciences) according to the manufacturer’s protocols. A modified MitoTOX™ OXPHOS Complex III Activity Kit (MitoSciences) was used to quantify Complex III activity. Results are presented as milli-optical density per minute (mOD/min). For the Complex I (NADH dehydrogenase) assay, 20 μg of mitochondrial extract from each mouse heart was applied to wells where the enzyme was immunocaptured. Enzyme activity was measured by monitoring the oxidation of NADH to NAD⁺, accompanied by the reduction of a dye that increases absorbance at 450 nm. Complex II (succinate-coenzyme Q reductase) was similarly immunocaptured in wells coated with anti-Complex II monoclonal antibody. Following this in-well purification, enzyme activity was assessed by coupling ubiquinol production with the reduction of 2,6-dichlorophenolindophenol (DCPIP), resulting in a decrease in absorbance at 600 nm. For the Complex III activity assay, 5 mg/mL of mouse heart mitochondrial sample was used in place of bovine heart mitochondria provided in the kit, with no additional drug treatments. Complex III activity was quantified by tracking the reduction of cytochrome c, observed as a linear increase in absorbance at 550 nm. To ensure specificity, rotenone and KCN were used as inhibitors for Complexes I and IV, respectively.

**1.21 Measurement of ATP**

Isolated hearts were homogenized in 2.5% (v/v) trichloroacetic acid (500 μL per 20 mg tissue). After centrifugation, 400 μL of the supernatant was neutralized with 80 μL of 1 M Tris. ATP concentration in the supernatant was then measured using a luciferin-luciferase assay and the ATP Bioluminescent Assay Kit. Soluble protein concentration was determined using the bicinchoninic acid (BCA) method following the manufacturer's protocol (Pierce BCA Protein Assay Kit, ThermoScientific). ATP levels were normalized to protein content and are reported as nanomoles of ATP per milligram of tissue protein.

**1.22 Statistical analysis**

In this study, the cellular and molecular experiments were independently repeated for at least 3 times. The animal assays involved were independently repeated for at least 6 mice. Data from replications were averaged and expressed as mean value ± standard deviation (SD). The statistical analysis was conducted by GraphPad Prism 5.0 (GraphPad Software, Inc., San Diego, CA, USA). Unpaired t-test was utilized to determine differences between two groups.  Analysis of variance (ANOVA) was performed for the comparison of multiple groups. Bonferroni post-hoc testing was used following ANOVA for analyzing all comparisons among groups. *P* < 0.05 was deemed as statistically significant.

1. Srinivasan K, Viswanad B, Asrat L, Kaul CL, Ramarao P. Combination of high-fat diet-fed and low-dose streptozotocin-treated rat: a model for type 2 diabetes and pharmacological screening. Pharmacological research 2005; 52:313-20.

2. Zhang N, Yu H, Liu T, Zhou Z, Feng B, Wang Y, et al. Bmal1 downregulation leads to diabetic cardiomyopathy by promoting Bcl2/IP3R-mediated mitochondrial Ca(2+) overload. Redox biology 2023; 64:102788.

3. Lu QB, Ding Y, Liu Y, Wang ZC, Wu YJ, Niu KM, et al. Metrnl ameliorates diabetic cardiomyopathy via inactivation of cGAS/STING signaling dependent on LKB1/AMPK/ULK1-mediated autophagy. Journal of advanced research 2023; 51:161-79.

4. Lu QB, Fu X, Liu Y, Wang ZC, Liu SY, Li YC, et al. Disrupted cardiac fibroblast BCAA catabolism contributes to diabetic cardiomyopathy via a periostin/NAP1L2/SIRT3 axis. 2023; 28:93.

5. Sun HJ, Xiong SP, Wu ZY, Cao L, Zhu MY, Moore PK, et al. Induction of caveolin-3/eNOS complex by nitroxyl (HNO) ameliorates diabetic cardiomyopathy. Redox biology 2020; 32:101493.

6. Yan D, Cai Y, Luo J, Liu J, Li X, Ying F, et al. FOXO1 contributes to diabetic cardiomyopathy via inducing imbalanced oxidative metabolism in type 1 diabetes. 2020; 24:7850-61.

7. Wang D, Green MF, McDonnell E, Hirschey MD. Oxygen flux analysis to understand the biological function of sirtuins. Methods in molecular biology (Clifton, NJ) 2013; 1077:241-58.

8. Kim HK, Ko TH, Song IS, Jeong YJ, Heo HJ, Jeong SH, et al. BH4 activates CaMKK2 and rescues the cardiomyopathic phenotype in rodent models of diabetes. 2020; 3.

9. Ansari M, Kurian GA. Hydrogen sulfide preconditioning could ameliorate reperfusion associated injury in diabetic cardiomyopathy rat heart through preservation of mitochondria. Biochimie 2019; 158:208-16.
