# Supplementary material for: Therapeutic Targeting of Decr1 Ameliorates Cardiomyopathy by Suppressing Mitochondrial Fatty Acid Oxidation in Diabetic Mice
Source: J Cachexia Sarcopenia Muscle. 2025 Mar 7;16(2):e13761. doi: 10.1002/jcsm.13761 (PMC11886612; doi:10.1002/jcsm.13761)
Supplement: Supplementary file 2 — Fig. S1 The expression paradigm of Decr1. (a) Decr1 is expressed in all organs throughout the body, with its expression being most pronounced in the cardiomyocytes in the HUMAN PROTEIN ATLAS database. (b) Decr1 expression levels in different cells of human heart. (c) Decr1 displayed the highest expression in the cardiomyocytes of the heart. (d) The immunohistochemical staining of Decr1 in the heart tissues from the HUMAN PROTEIN ATLAS database. (e) Decr1 was mainly expressed in cardiomyocytes using the Tabula Muris database. Figure S2. Decr1 expression is elevated in the heart of T2DM mice and HG/HP‐exposed cardiomyocytes. (a) The protein expression of Decr1 in the heart of control and T2D mice. (b) Immunohistochemistry of Decr1 in the heart. (c) Immunofluorescence showing the co‐localization of Decr1 and cardiomyocyte marker. (d) Relative mRNA level of Decr1 in isolated cardiomyocytes, cardiac fibroblasts, and cardiac endothelial cells. (e) The protein expression of Decr1 in HG/HP‐exposed cardiomyocytes. (f) Relative mRNA level of Decr1 in HG/HP‐exposed cardiomyocytes. Data were calculated as means ± SD. *p < 0.05 vs. Control (Con) or normal glucose (NG). Figure S3. Cardiac‐specific deficiency of Decr1 alleviates DCM in T2D mice. (a) The protein expression of Decr1. (b) EF and FS. (c) Representative images of Sirius Red staining and TUNEL staining. (d) The quantification of cardiac fibrosis measured by Sirius Red staining. (e) The quantitative analysis of TUNEL staining. (f) The activity of Complex I, Complex II, Complex III, and Complex IV. (g) ATP contents. Data were calculated as means ± SD. *p < 0.05 vs. ShCon. †p < 0.05 vs. T2D + ShCon. Figure S4. Cardiac‐specific overexpression of Decr1 aggravates DCM in T2D mice. (a) The flow chart of animal experiments. (b) The protein expression of Decr1. (c) Serum LDH level and CK‐MB level. (d) EF and FS. (e) Representative images of HE staining, WGA staining, Sirius Red staining, TUNEL staining and DHE staining. (f) Card [file JCSM-16-e13761-s002.docx]

**Therapeutic targeting of Decr1 ameliorates cardiomyopathy by suppressing mitochondrial fatty acid oxidation in diabetic mice**

Qing-Bo Lu^1#^, He-Ting Sun^2#^, Kuo Zhou^3#^, Jia-Bao Su^4#^, Xin-Yu Meng^5^, Guo Chen^5^, Ao-Yuan Zhang^5^, An-Jing Xu^5^, Chen-Yang Zhao^5^, Yuan Zhang^5^, Yao Wang^5^, Hong-Bo Qiu^5^, Zhuo-Lin Lv^5^, Zheng-Yang Bao^6^, Jian Zhu^1*^, Feng Xiao^7*^, Xue-Xue Zhu^1,5*^, Hai-Jian Sun^1,5,8*^

^1^Department of Endocrinology, Affiliated Hospital of Jiangnan University, Jiangnan University, Wuxi, 214125, Jiangsu, China.

^2^School of Pharmacy, Collaborative Innovation Center of Advanced Drug Delivery System and Biotech Drugs in Universities of Shandong, Key Laboratory of Molecular Pharmacology and Drug Evaluation (Yantai University), Ministry of Education, Yantai University, Yantai, 264005, China

^3^Department of Cardiology, The First Affiliated Hospital of Nanjing Medical University, Nanjing 210029, China.

^4^Department of Anesthesiology, Affiliated Hospital of Jiangnan University, Jiangnan University, Wuxi 214122, China.

^5^MOE Medical Basic Research Innovation Center for Gut Microbiota and Chronic Diseases, School of Medicine, Jiangnan University, Wuxi, 214122, China.

^6^Research Institute for Reproductive Health and Genetic Diseases, Wuxi Maternity and Child Health Care Hospital, Wuxi 214125, China.

^7^Department of Cardiology, the Affiliated Wuxi People's Hospital of Nanjing Medical University, Wuxi People's Hospital, Wuxi Medical Center, Nanjing Medical University, Wuxi 214023, China

^8^State Key Laboratory of Natural Medicines, China Pharmaceutical University, No. 24 Tongjia Lane, Nanjing 210009, China.

^#^Contributed equally.

**Running title:** Decr1 in diabetic cardiomyopathy

**Figures and Tables**: 8 main Figures, 16 supplementary figures, 8 Tables

*Address for correspondence:

Jian Zhu, Email: drzhujian@hotmail.com; Feng Xiao, Email: xiaofeng@njmu.edu.cn; Xue-Xue Zhu, Email: [zhuxuexue117@163.com](mailto:zhuxuexue117@163.com); Hai-Jian Sun, Email: haijian.sun@jiangnan.edu.cn; Phone/Fax: 0510-85328363

**Supplementary data**


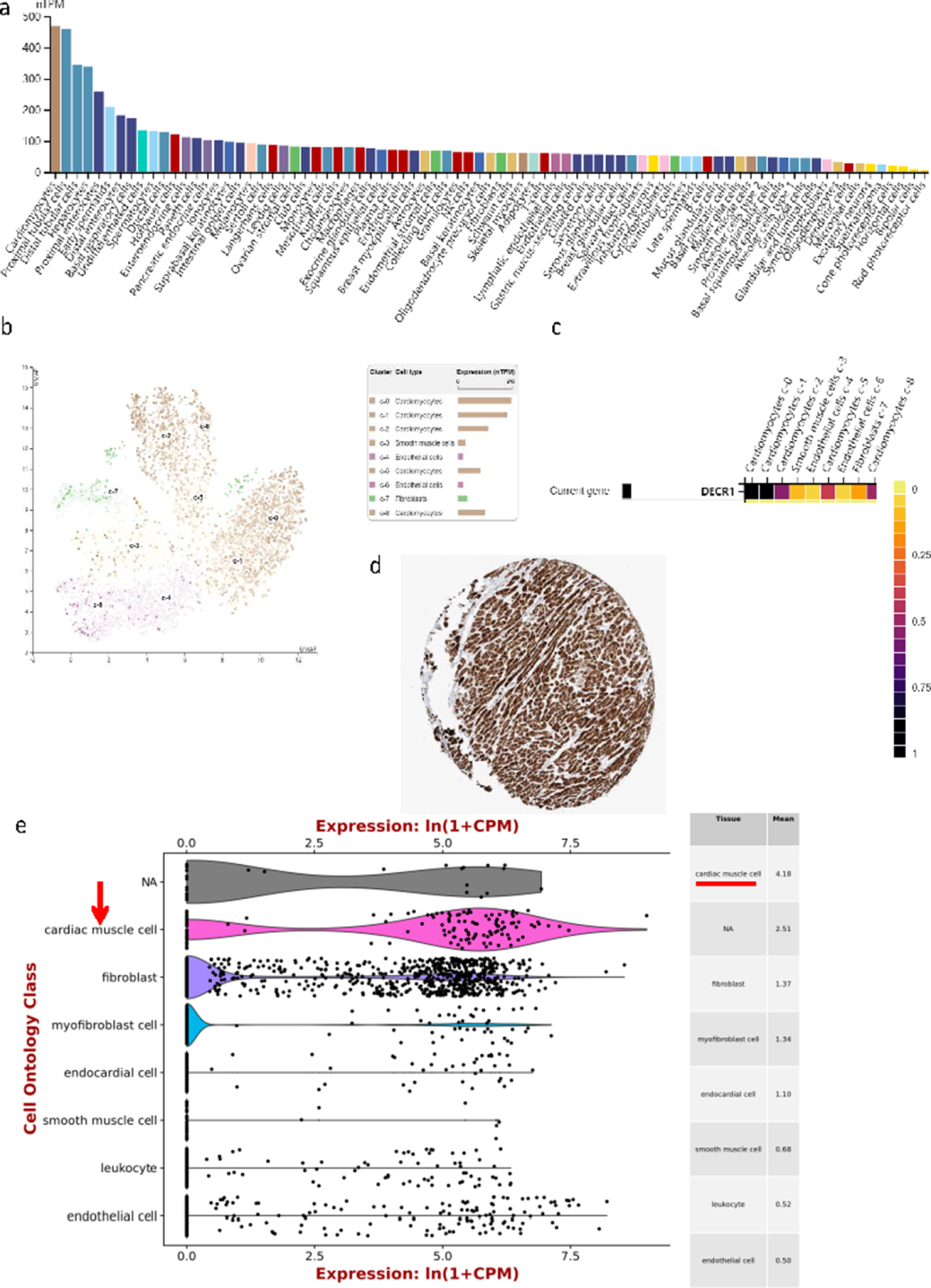


**Fig. S1.** **The expression paradigm of Decr1.**

(**a**) Decr1 is expressed in all organs throughout the body, with its expression being most pronounced in the cardiomyocytes in the HUMAN PROTEIN ATLAS database. (**b**) Decr1 expression levels in different cells of human heart. (**c**) Decr1 displayed the highest expression in the cardiomyocytes of the heart. (**d**) The immunohistochemical staining of Decr1 in the heart tissues from the HUMAN PROTEIN ATLAS database. (**e**) Decr1 was mainly expressed in cardiomyocytes using the Tabula Muris database.

**
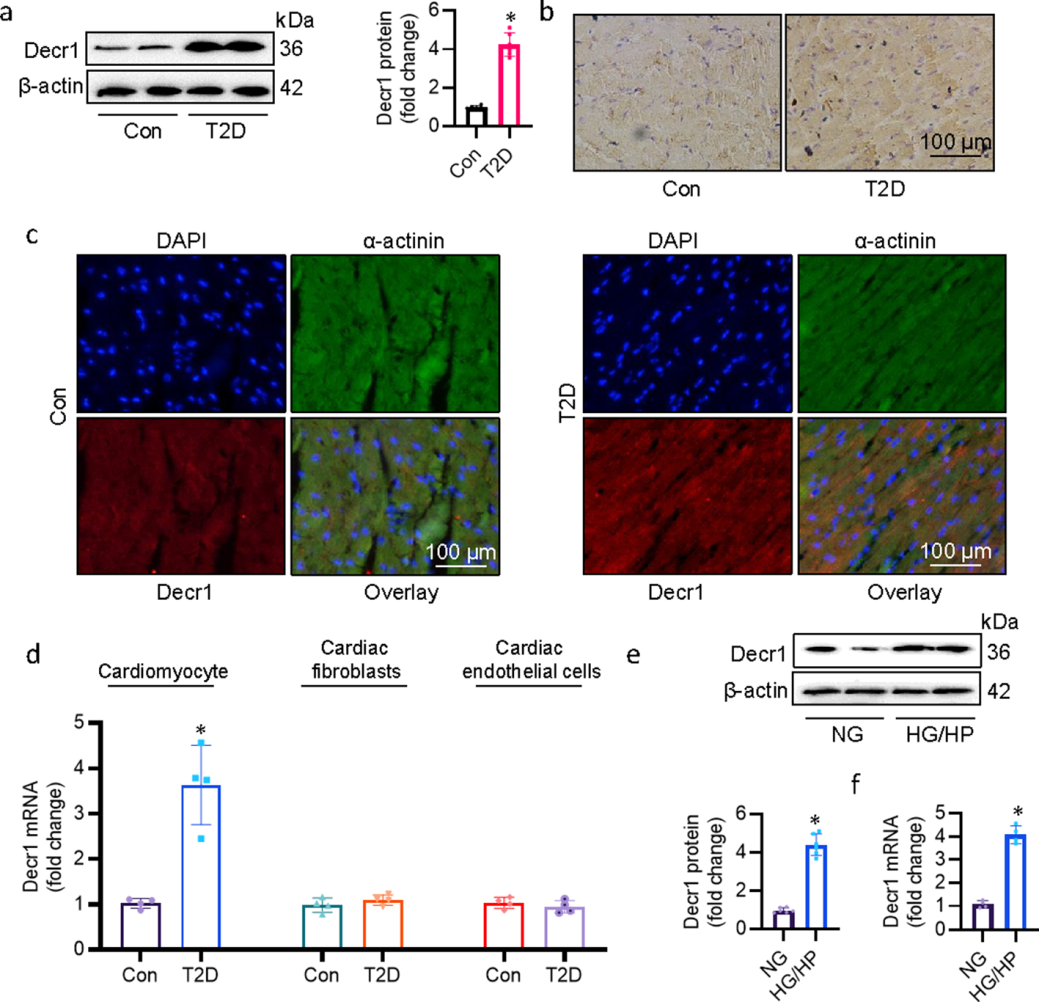
**

**Fig. S2.** **Decr1 expression is elevated in the heart of T2DM mice and HG/HP-exposed cardiomyocytes.**

(**a**) The protein expression of Decr1 in the heart of control and T2D mice. (**b**) Immunohistochemistry of Decr1 in the heart. (**c**) Immunofluorescence showing the co-localization of Decr1 and cardiomyocyte marker. (**d**) Relative mRNA level of Decr1 in isolated cardiomyocytes, cardiac fibroblasts, and cardiac endothelial cells. (**e**) The protein expression of Decr1 in HG/HP-exposed cardiomyocytes. (**f**) Relative mRNA level of Decr1 in HG/HP-exposed cardiomyocytes. Data were calculated as means ± SD. *P < 0.05 *vs*. Control (Con) or normal glucose (NG).

**
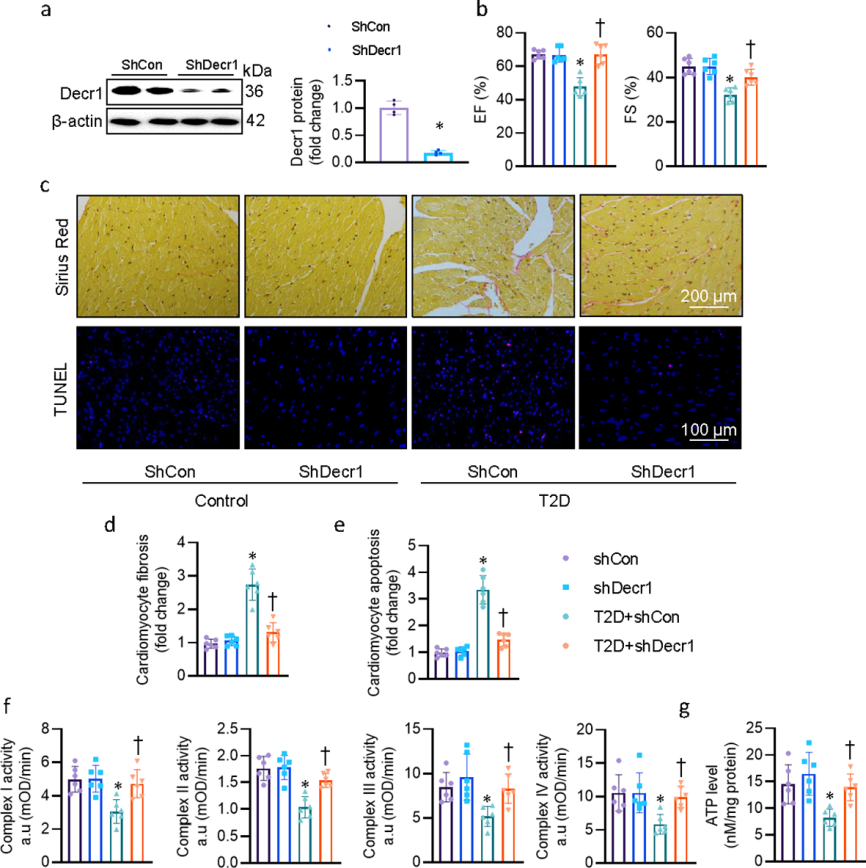
**

**Fig. S3. Cardiac-specific deficiency of Decr1 alleviates DCM in T2D mice.**

(**a**) The protein expression of Decr1. (**b**) EF and FS. (**c**) Representative images of Sirius Red staining and TUNEL staining. (**d**) The quantification of cardiac fibrosis measured by Sirius Red staining. (**e**) The quantitative analysis of TUNEL staining. (f) The activity of Complex I, Complex II, Complex III, and Complex IV. (**g**) ATP contents. Data were calculated as means ± SD. *P < 0.05 *vs*. ShCon. †P < 0.05 *vs*. T2D+ShCon.


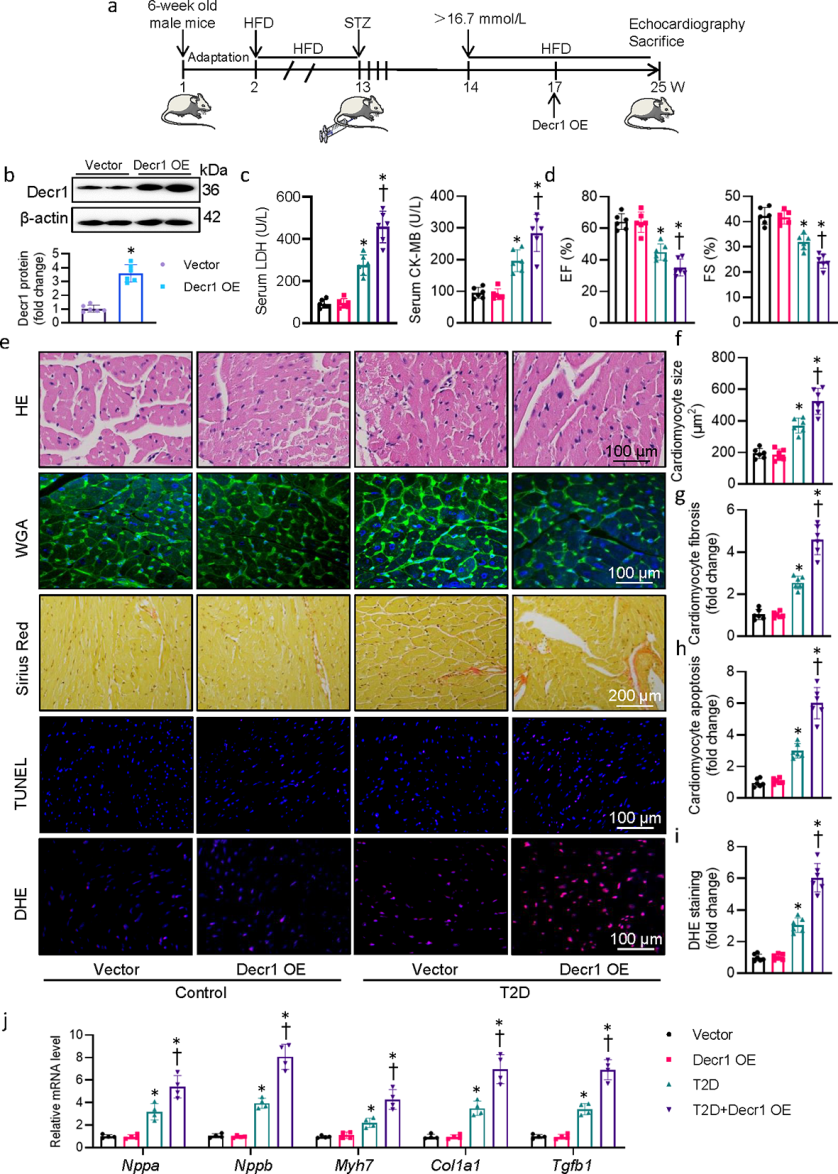


**Fig. S4.** **Cardiac-specific overexpression of Decr1 aggravates DCM in T2D mice.**

(**a**) The flow chart of animal experiments. (**b**) The protein expression of Decr1. (**c**) Serum LDH level and CK-MB level. (**d**) EF and FS. (**e**) Representative images of HE staining, WGA staining, Sirius Red staining, TUNEL staining and DHE staining. (**f**) Cardiomyocyte size analyzed by WGA staining of left ventricle myocardium. At least 100 cells measured in different visual fields from 6 samples per group. Each point on each column represents the average area of 100 myocardial cells in each sample. The quantification of average cross-sectional area of cardiomyocytes compared with control in the indicated groups was shown in the bar graph. (**g**) The quantitative analysis of cardiac fibrosis. (**h**) The quantitative analysis of TUNEL staining. (**i**) The quantitative analysis of DHE staining. (**j**) Relative mRNA level of *Nppa*, *Nppb, Myh7, Col1a1*, and *Tgfb1*. Data were calculated as means ± SD. *P < 0.05 *vs*. Vector. †P < 0.05 *vs*. T2D.


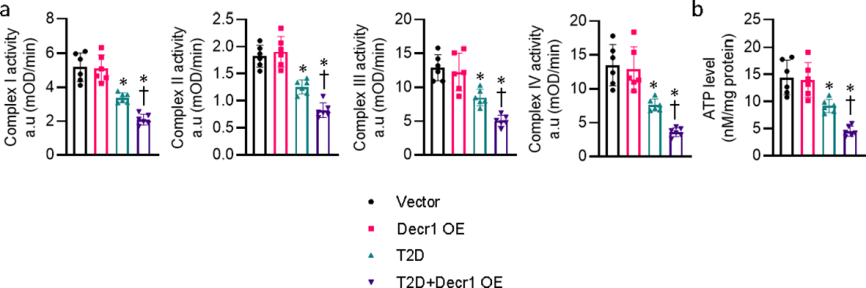


**Fig. S5. Cardiac-specific overexpression of Decr1 aggravates mitochondrial damage in the heart of DCM mice.** (a) The activity of Complex I, Complex II, Complex III, and Complex IV. (**b**) ATP contents. Data were calculated as means ± SD. *P < 0.05 *vs*. Vector. †P < 0.05 *vs*. T2D.


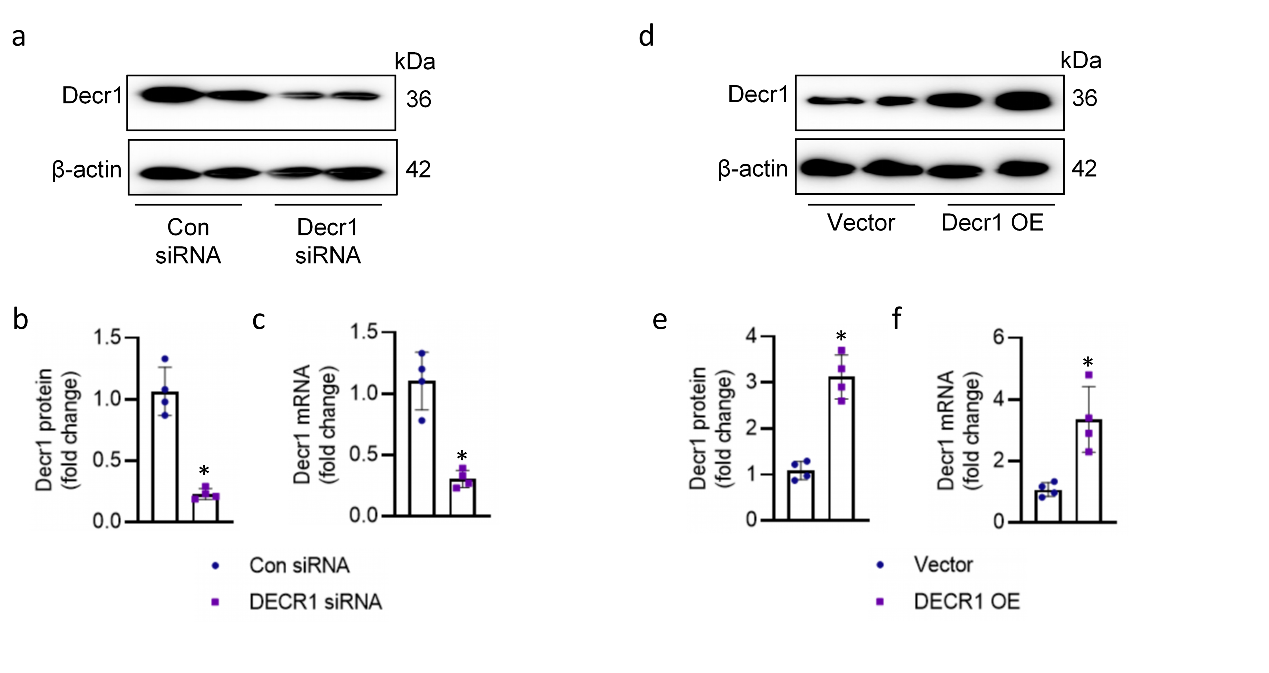


**Fig. S6.** **The protein and mRNA expression levels of Decr1 in cardiomyocytes *in vitro***

(**a, b**) The protein expression of Decr1 in cardiomyocytes after Decr1 knockdown. (**c**) The mRNA level of Decr1 in cardiomyocytes after Decr1 knockdown. (**d, e**) The protein expression of Decr1 in cardiomyocytes after Decr1 overexpression. (**f**) The mRNA level of Decr1 in cardiomyocytes after Decr1 overexpression. Data were calculated as means ± SD. *P < 0.05 *vs*. Con siRNA or Vector.


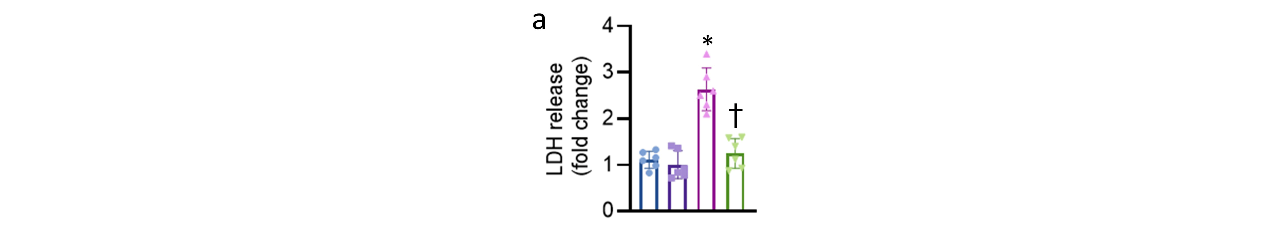


**Fig. S7.** **Downregulation of Decr1 inhibited HG/HP-induced injury** **in cardiomyocytes *in vitro***

(**a**) LDH release. Data were calculated as means ± SD. *P < 0.05 *vs*. Con siRNA. †P < 0.05 *vs*. HG/HP+Con siRNA.


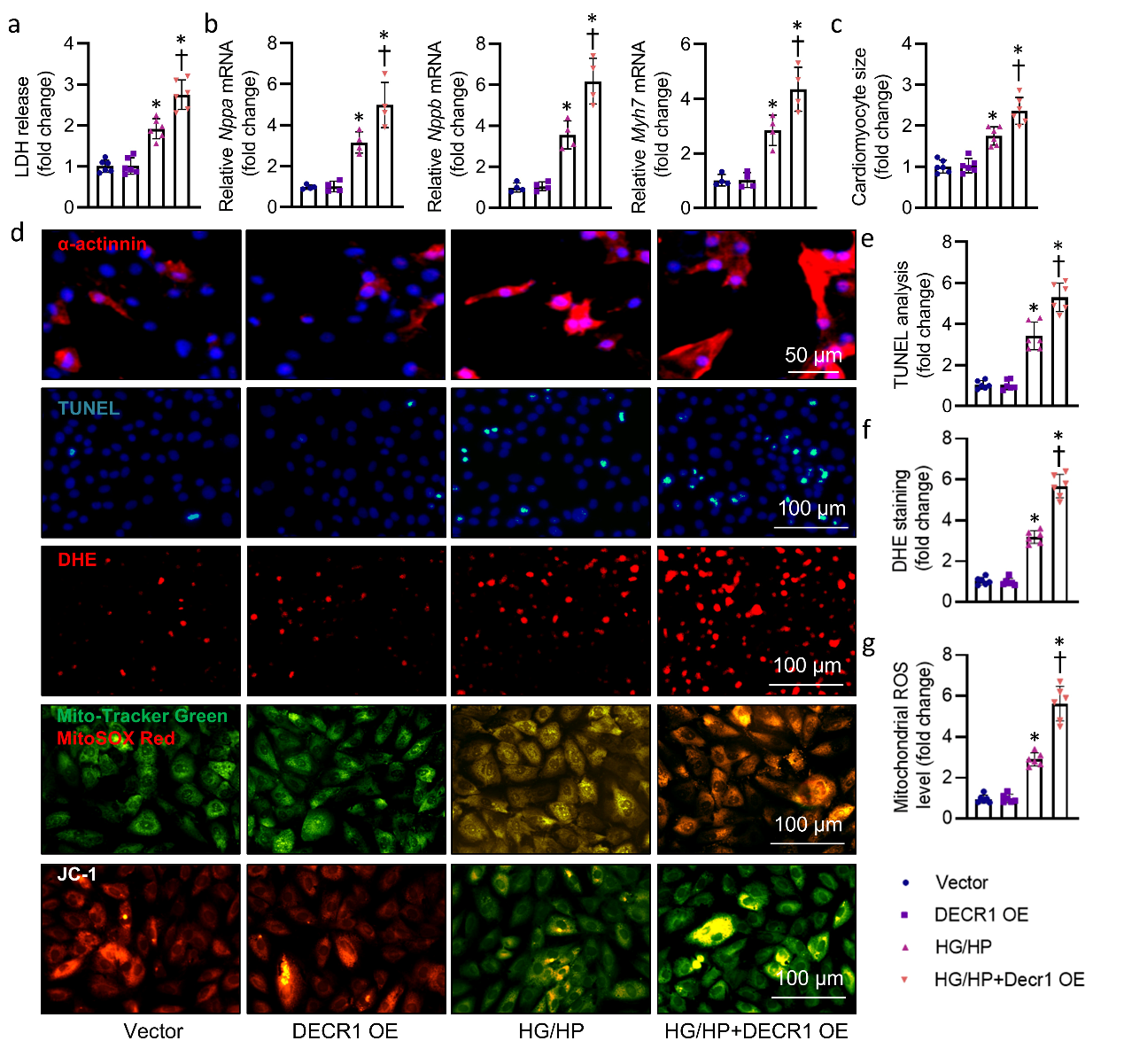


**Fig. S8.** **Upregulation of Decr1 worsens HG/HP-induced injury in cardiomyocytes**

(**a**) LDH release. (**b**) Relative mRNA level of *Nppa*, *Nppb, Myh7*. (**c**) Cardiomyocyte size measured by α-actinin staining. (**d**) Representative images of α-actinin staining, TUNEL staining, DHE staining, mitochondrial ROS levels, and JC-1 staining. (**e**) Quantification of TUNEL staining. (**f**) Quantification of DHE staining. (**g**) Quantification of mitochondrial ROS levels. Data were calculated as means ± SD. *P < 0.05 *vs*. Vector. †P < 0.05 *vs*. HG/HP.


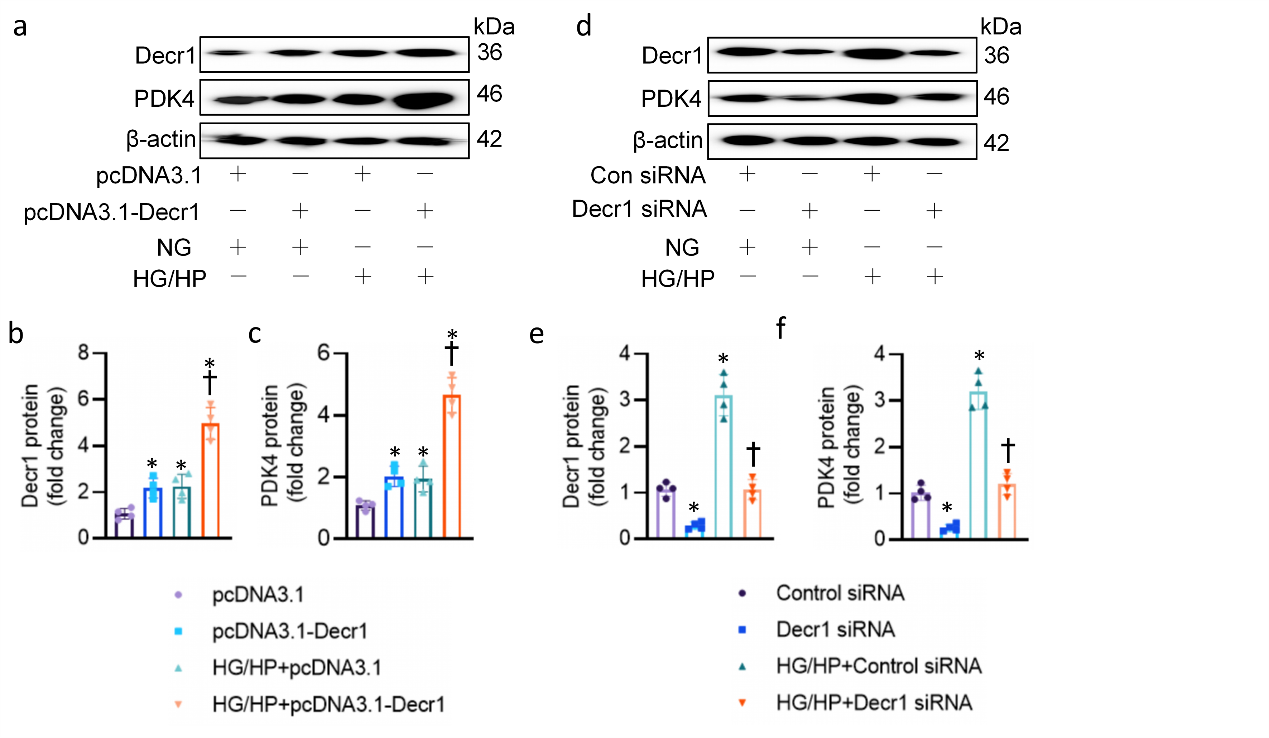


**Fig. S9.** **Effects of Decr1 on the protein expression of PDK4 in cardiomyocytes**

(**a-c**) The protein expression of PDK4 and Decr1 in cardiomyocytes after overexpression of Decr1. (**d-f**) The protein expression of PDK4 and Decr1 in cardiomyocytes after knockdown of Decr1. Data were calculated as means ± SD. *P < 0.05 *vs*. pcDNA3.1 or Con siRNA. †P < 0.05 *vs*. HG/HP+pcDNA3.1 or HG/HP+Con siRNA.

**
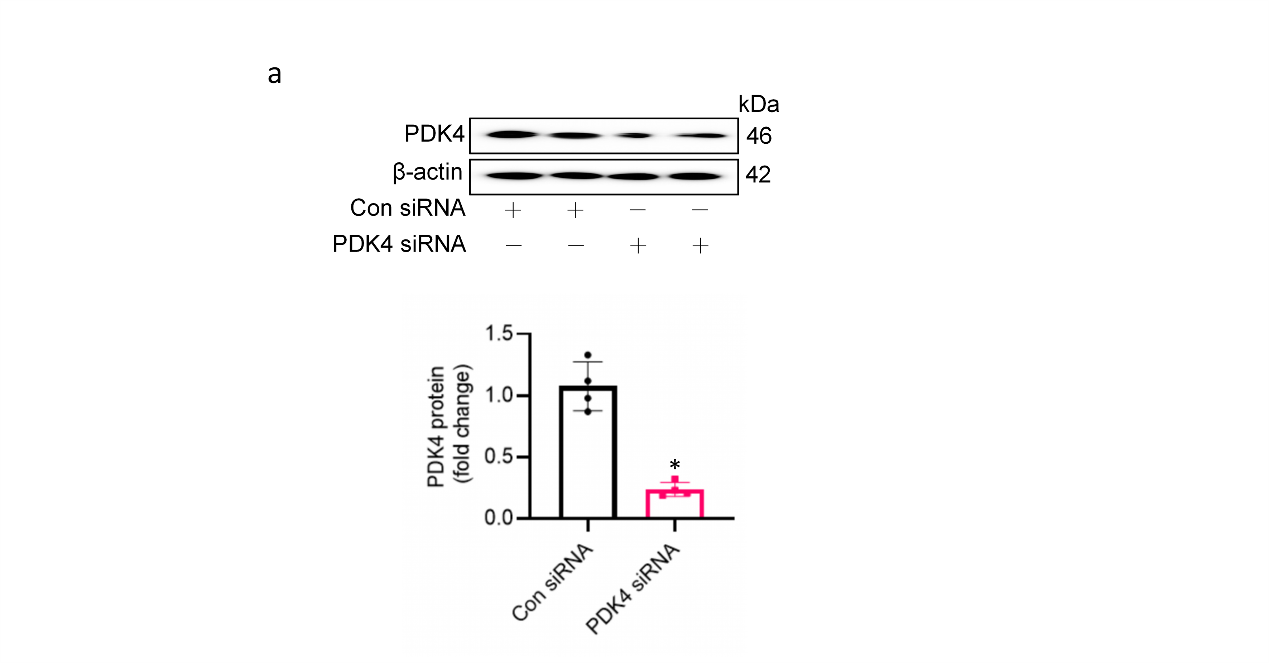
**

**Fig. S10.** **The protein expression of PDK4 after transfection of PDK4 siRNA**

Data were calculated as means ± SD. *P < 0.05 *vs*. Con siRNA.


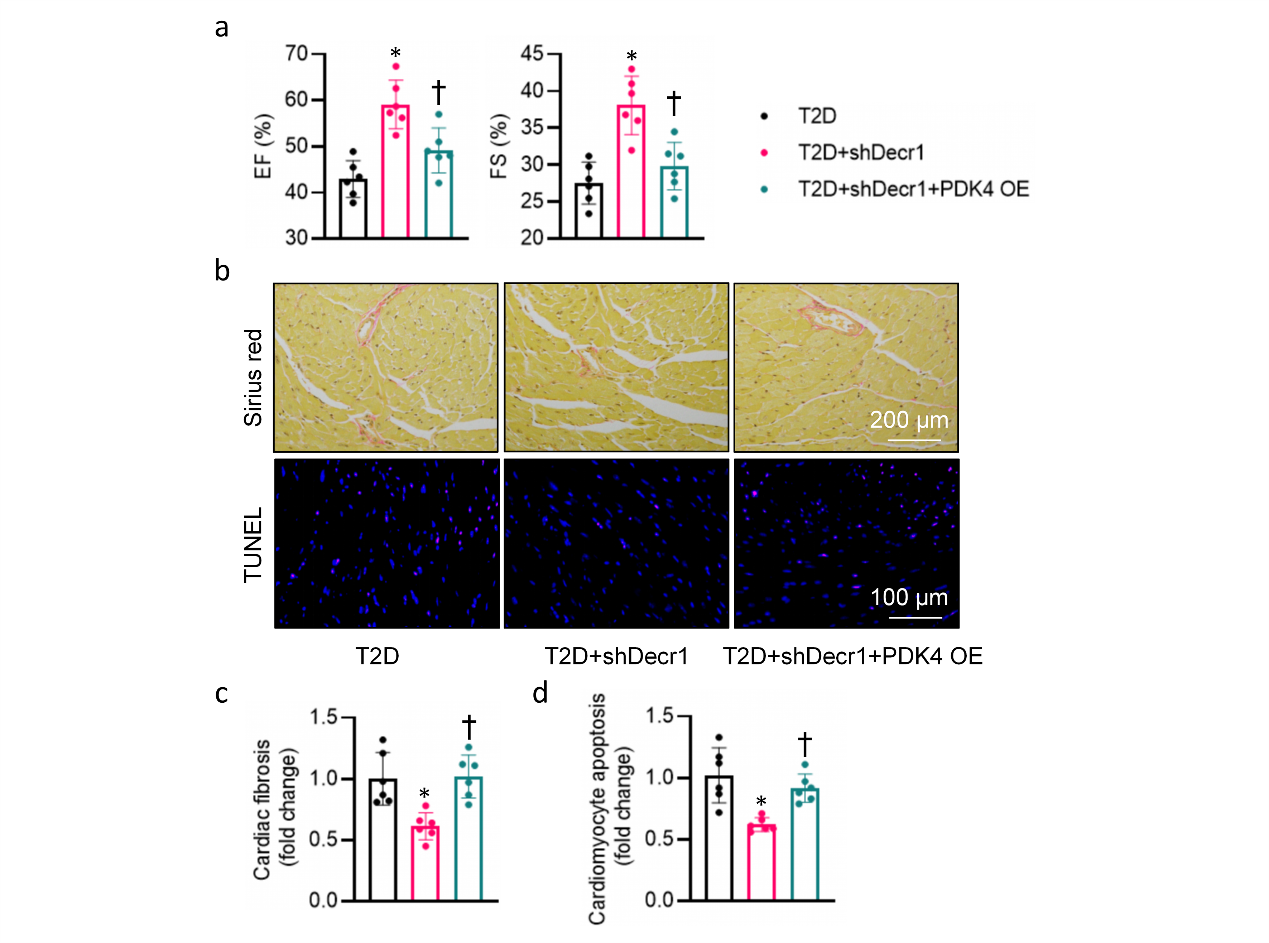


**Fig. S11.** **Overexpression of PDK4 blocked the benefits of Decr1 knockdown in DCM.** (**a**) EF and FS. (**b**) Sirius Red staining, and TUNEL staining of left ventricle myocardium. (**c**) Representative images of Sirius Red staining and TUNEL staining. (**d**) The quantification of cardiac fibrosis measured by Sirius Red staining. (**e**) The quantitative analysis of TUNEL staining. Data were calculated as means ± SD. *P < 0.05 *vs*. T2D. †P < 0.05 *vs*. T2D+ShDecr.


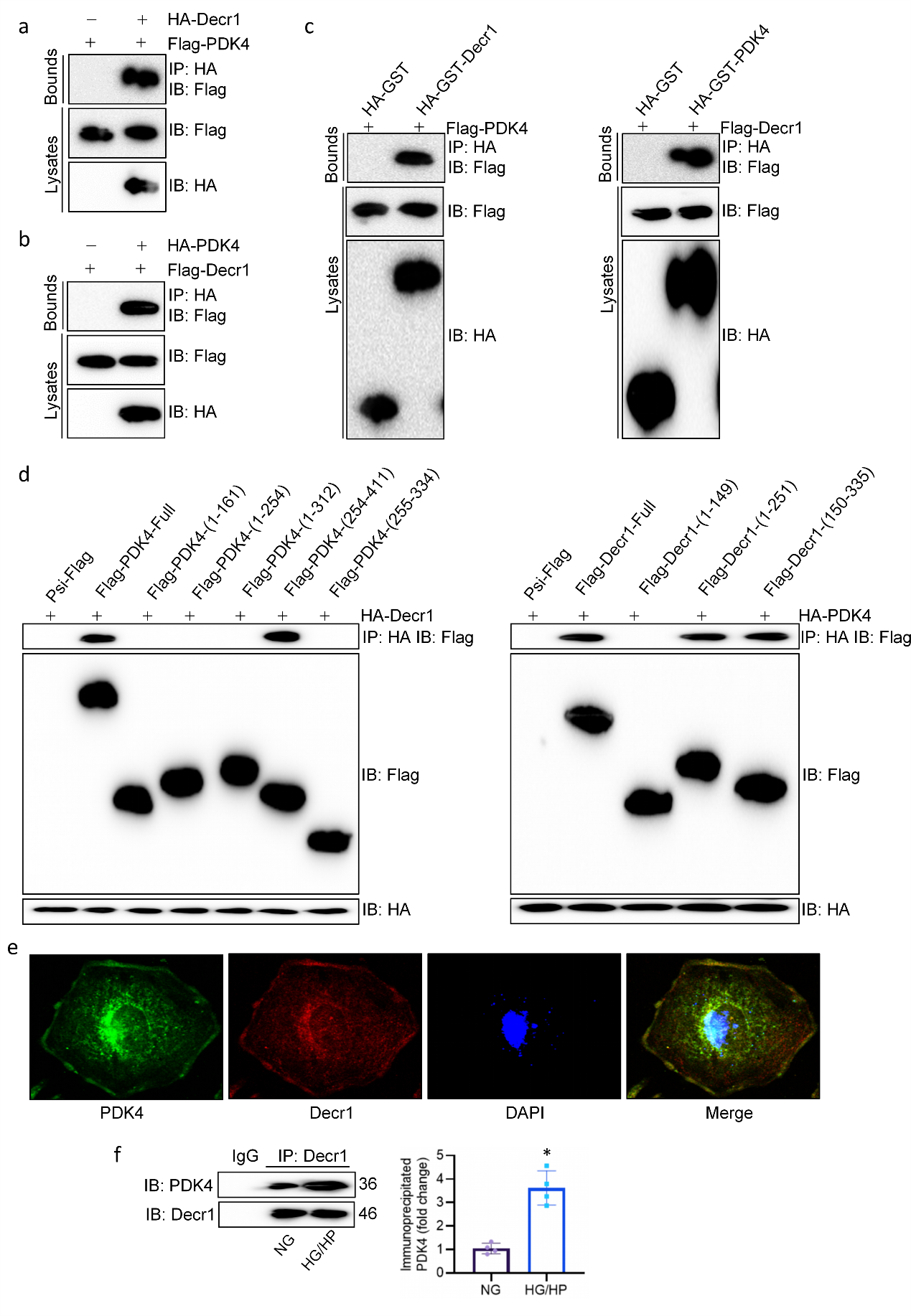


**Fig. S12. The direct interaction of Decr1 and PDK4.** (**a**) The co-IP assays in HEK293 cells transfected with Flag-tagged PDK4 and HA-tagged Decr1. Anti-Flag and anti-HA antibodies were used as western blot probes. (**b**) The co-IP assays in HEK293 cells transfected with Flag-tagged Decr1 and HA-tagged PDK4. Anti-Flag and anti-HA antibodies were used as western blot probes. (**c**) GST precipitation assays showing direct PDK4-Decr1 binding. Purified GST was used as a control. (**d**) Results from co-IP assays showing the binding regions between Decr1 and PDK4. (**e**) The co-localization of PDK4 and Decr1 assessed by laser confocal microscope. (**f**) The interaction of PDK4 with Decr1 in cardiomyocytes upon exposure of HG/HP. Data were calculated as means ± SD. *P < 0.05 *vs*. NG.

**
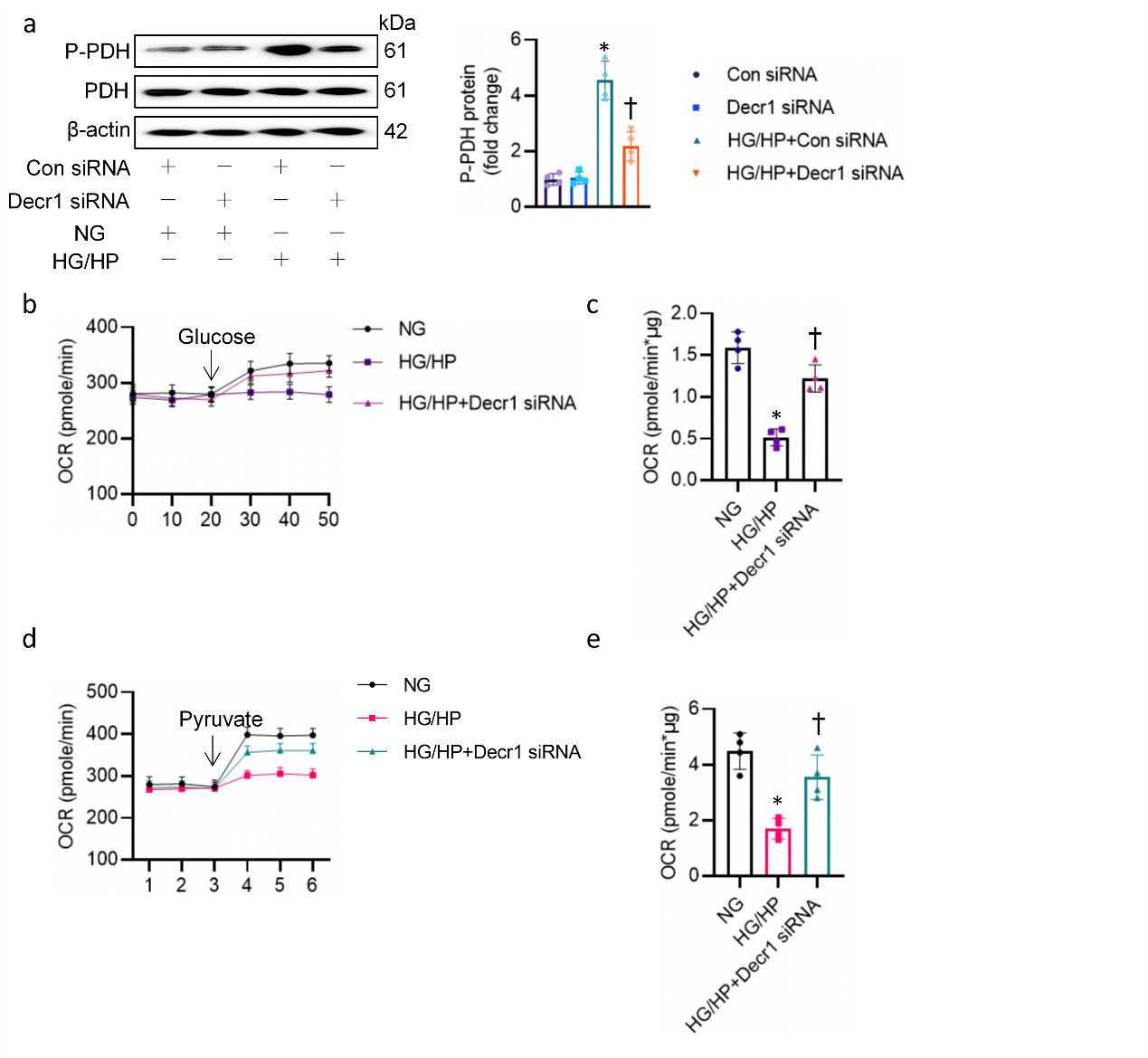
**

**Fig. S13.** **Suppression of Decr1 P-PDH expression and restored glucose oxidation in diabetic cardiomyocytes.** (**a**) The phosphorylation level of PDH. (**b**) Kinetic oxygen consumption rate (OCR) responses of isolated cardiomyocytes to 10 mM glucose. (**c**) Calculated glucose oxidation rate. (**d**) Kinetic OCR responses of isolated cardiomyocytes to 1 mM pyruvate. (**e**) Calculated pyruvate oxidation rate. The glucose or pyruvate oxidation rate was calculated by the OCR increase. Data were calculated as means ± SD. *P < 0.05 *vs*. Con siRNA or NG. †P < 0.05 *vs*. HG/HP+Con siRNA or HG/HP.


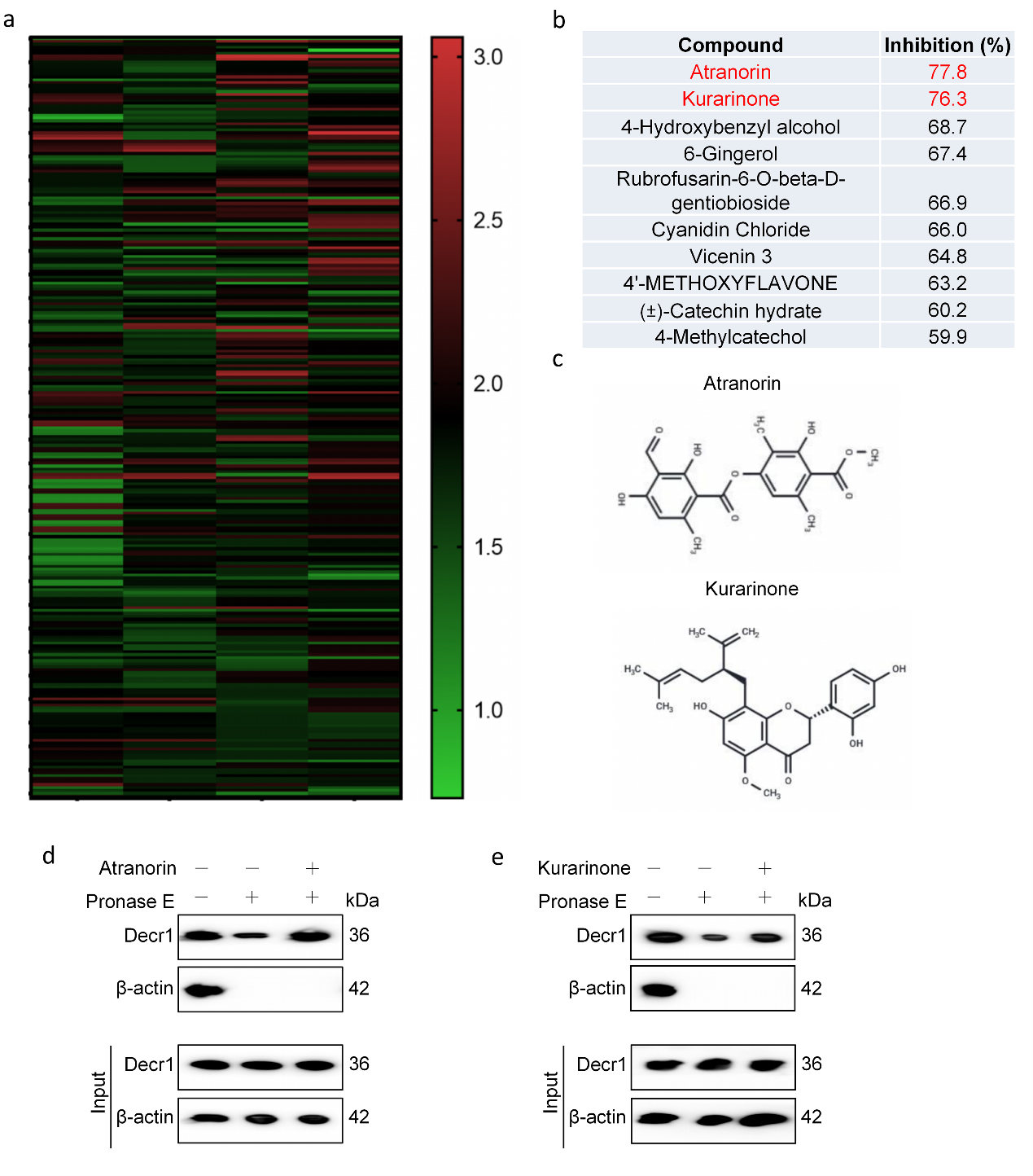


**Fig. S14.** **Atranorin and Kurarinon show strong ability to inhibit Decr1 activity.** (**a**) Heatmap showing the effects of different compounds on the activity of Decr1. (**b**) Top ten compounds showed the suppressive effects on the activity of Decr1. (**c**) Chemical structure of Atranorin and Kurarinon. (**d**) DARTS showing the direct binding of Atranorin to Decr1 protein. (**e**) DARTS showing the direct binding of Kurarinon to Decr1 protein.


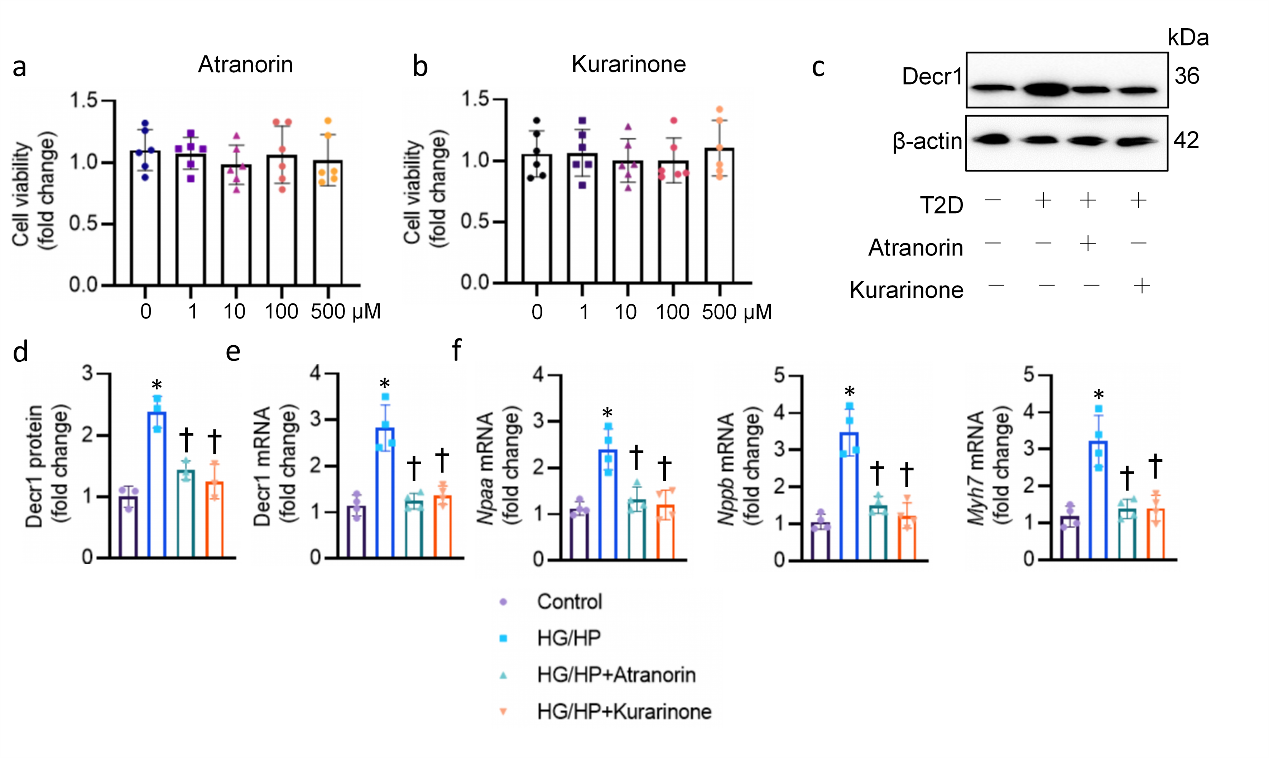


**Fig. S15. Atranorin and Kurarinon protect cardiomyocytes against HG/HP-induced injury.** (**a**) Cell viability after treatment with Atranorin. (**b**) Cell viability after treatment with Kurarinon. (**c, d**) The protein expression of Decr1. (**e**) The mRNA level of Decr1. (**f**) Relative mRNA level of *Nppa*, *Nppb, Myh7*. Data were calculated as means ± SD. *P < 0.05 *vs*. Control. †P < 0.05 *vs*. HG/HP.


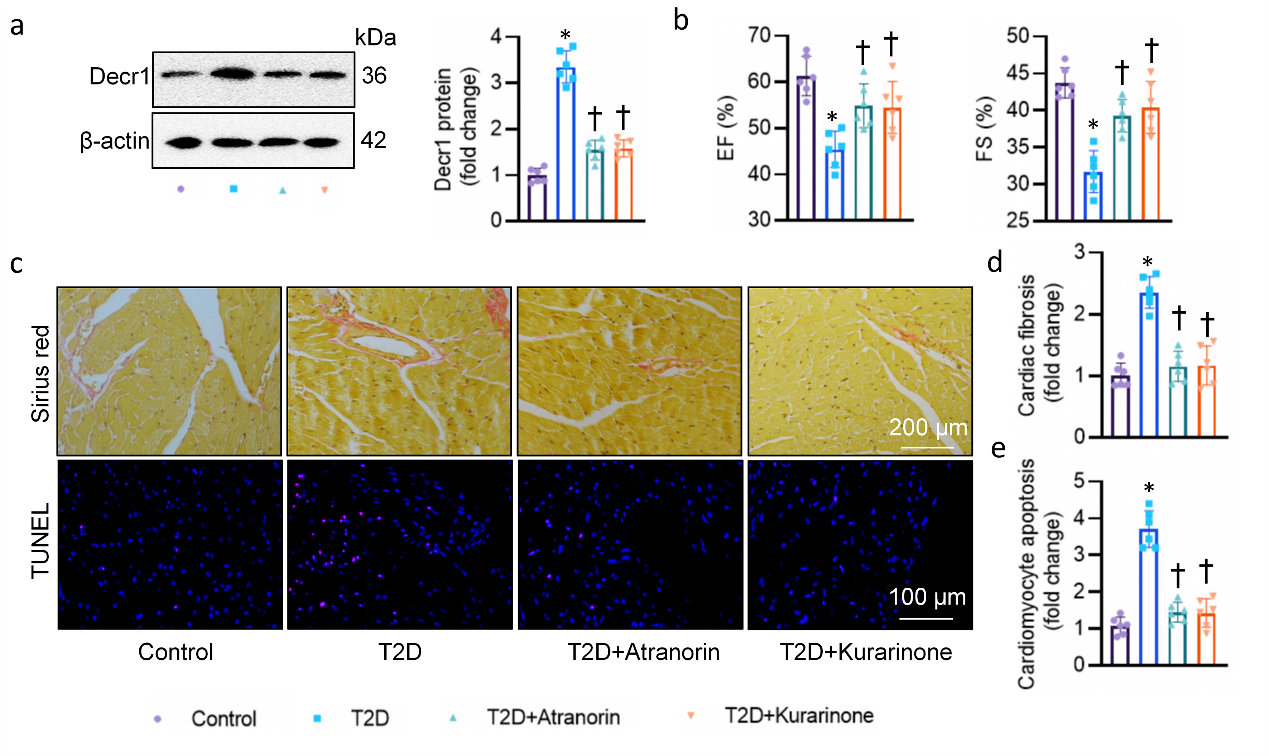


**Fig. S16.** **Atranorin and Kurarinon improved DCM by binding to and suppressing Decr1.** (**a**) The protein expression of Decr1. (**b**) EF and FS. (**c**) Representative images of Sirius Red staining and TUNEL staining. (**d**) The quantification of cardiac fibrosis measured by Sirius Red staining. (**e**) The quantitative analysis of TUNEL staining. Data were calculated as means ± SD. *P < 0.05 *vs*. Control. †P < 0.05 *vs*. T2D.


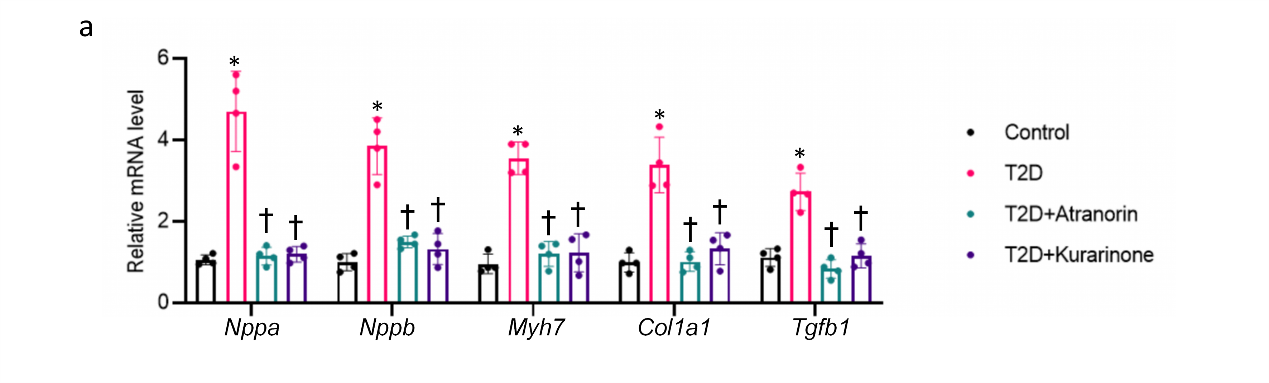


**Fig. S17. Atranorin and Kurarinon prevented cardiac hypertrophy and fibrosis in T2D mice.** Relative mRNA level of *Nppa*, *Nppb, Myh7, Col1a1*, and *Tgfb1*. Data were calculated as means ± SD. *P < 0.05 *vs*. Control. †P < 0.05 *vs*. T2D.

**Table S1: Table S1. Information for primary and secondary antibodies.**

| Target | Company | Cat. No. | Dilution ratio |
| --- | --- | --- | --- |
| Decr1  PDK4  β-MyHc  β-actin  IL-1β | Biorbyt  Proteintech  Abcam  Abcam  Abcam | orb556625  12949-1-AP  Ab207926  ab7817  ab283818 | 1: 1,000 for WB  1:1,000 for WB  1: 1,000 for WB  1: 1,000 for WB  1: 1,000 for WB |
| Cleaved  Caspase-3 | CST | #9661 | 1: 1,000 for WB |
| PDK4 | Abcam | ab110336 | 1: 1,000 for IF |
| CD36 | Abcam | ab252922 | 1: 1,000 for WB |
| CPT1α | Proteintech | 15184-1-AP | 1: 1,000 for WB |
| HADHA | Biorbyt | orb1274453 | 1: 1,000 for WB |
|  |  |  |  |
| Acetylated-Lysine | CST | #9441 | 1: 1,000 for WB |
| HDAC3 | CST | #85057 | 1: 1,000 for WB |
| P-HDAC3 | CST | #3815 | 1: 1,000 for WB |
| COX4 | CST | #4850 | 1: 1,000 for WB |
| HA | Proteintech | 51064-2-AP | 1: 1,000 for WB |
| Flag | Proteintech | 66008-4-Ig | 1: 1,000 for WB |
| PDH | CST | #2784 | 1: 1,000 for WB |
| P-PDH | CST | #31866 | 1: 1,000 for WB |
| α-actinin | Abcam | ab68194 | 1: 200 for IF |
| α-actinin | Abcam | #69758 | 1: 200 for IF |
| Goat Anti-Rabbit IgG H&L (Alexa Fluor® 594) | Abcam | ab150080 | 1: 1000 for IF |
| Goat Anti-Mouse IgG H&L (Alexa Fluor® 488) | Abcam | ab150113 | 1: 1000 for IF |
| Anti-mouse HRP secondary antibody | Immunoway | RS0001 | 1:5,000 |
| Anti-rabbit HRP secondary antibody  [Goat Anti-Rabbit IgG H&L (Alexa Fluor® 488)](https://www.abcam.cn/products/secondary-antibodies/goat-rabbit-igg-hl-alexa-fluor-488-ab150077.html)  [Goat Anti-Mouse IgG H&L (Alexa Fluor® 594)](https://www.abcam.cn/products/secondary-antibodies/goat-rabbit-igg-hl-alexa-fluor-488-ab150077.html) | Immunoway  Abcam  Abcam | RS0002  [ab150077](https://www.abcam.cn/products/secondary-antibodies/goat-rabbit-igg-hl-alexa-fluor-488-ab150077.html)  ab150116 | 1:5,000  1:1000  1: 1000 for IF |

**Table S2. Primers for Real-time quantitative PCR analysis in mice**.

| **Gene name** | **Sequences** |
| --- | --- |
| Mouse *Decr1* | 5′-GATCCGGGTCCTCAGAGGTTT-3′  5′-ATCAGGTGGTAGCATAGGCTT-3′ |
| Mouse *nppa* | 5′-ACCTGCTAGACCACCTGGAG-3′  5′-CCTTGGCTGTTATCTTCGGTACCGG-3′ |
| Mouse *nppb* | 5′-GAGGTCACTCCTATCCTCTGG-3′  5′-GCCATTTCCTCCGACTTTTCTC-3′ |
| Mouse *myh7* | 5'-CCGAGTCCCAGGTCAACAA-3'  5'-CTTCACGGGCACCCTTGGA-3' |
| Mouse *Cpxm2* | 5′-GGGCGCTATTATGGGCATCC-3′  5′-GGCCTTCTTGGGAGTTCTGT-3′ |
| Mouse *Oxct1* | 5′-GCCCTGCATAAGGGGTGTG-3′  5′-GCAAGGTTGCACCATTAGGAAT-3′ |
| Mouse *Cpt1* | 5′-CGGAGACGACGCTTTCGAC-3′  5′-CGTAGTTGGAAGTACACCAGGA-3′ |
| Mouse *Ucp2* | 5′-ATGGTTGGTTTCAAGGCCACA-3′  5′-TTGGCGGTATCCAGAGGGAA-3′ |
| Mouse *Acot1* | 5′-CCCCTGTGACTATCCTGAGAA-3′  5′-CAAACACTCACTACCCAACTGT-3′ |
| Mouse *Acot2* | 5′-GTTGTGCCAACAGGATTGGAA-3′  5′-GCTCAGCGTCGCATTTGTC-3′ |
| Mouse *Col1a1* | 5′-CCCAAGGAAAAGAAGCACGTC-3′  5′-AGGTCAGCTGGATAGCGACATC-3′ |
| Mouse *Tgfb1* | 5′-ATGTCACGGTTAGGGGCTC-3′  5′-GGCTTGCATACTGTGCTGTATAG-3′ |
| Mouse *PDK4* | 5′-CCGCTTAGTGAACACTCCTTC-3′  5′-TGACCAGCGTGTCTACAAACT-3′ |
| Mouse *Cyp4f15* | 5′-AGCTTCGGTAGCCCTAAAGGA-3′  5′-GCAGGTGTCAACATGCGAC-3′ |
| Mouse *Cdh26* | 5′-GTCATTACCGCAGGGACAGAG-3′  5′-CCCAACCAGTTTAGGAAAGGG-3′ |
| Mouse *Lrrc30* | 5′-CTGAGCATGTCGCACAACTG-3′  5′-CTAGGCGATTGGAGCCCAC-3′ |
| Mouse *Serpina3m* | 5′-CCTGTCGGTCTGCACTTCTT-3′  5′-ACTGCCTGAGGATCCAGCTA-3′ |
| Mouse *Gabra1* | 5′-AGACCGACATTTTCGTCACCA-3′  5′-GTTTAGCCGGAGCACTGTCAT-3′ |
| Mouse *Wdr93* | 5′-TGTTCCTATCGACGAAATGGGT-3′  5′-CTGCTTGCTGCTATCATCCAC-3′ |
| Mouse *Doc2b* | 5′-GACATGGTCCGAAAGACCCTG-3′  5′-CACCCGAGTCTCTCCAATGAA-3′ |
| Mouse *Serpina3k* | 5′-AGAGGAGCTAAACCTGCCCAA -3′  5′-ATACGGCCTTACGAATGCCAC-3′ |
| Mouse *Cxcl3* | 5′-ACATCCAGAGCTTGACGGTG-3′  5′-CTACCCCAGCTGCCATCAAA-3′ |
| Mouse *Mctp2* | 5′-CACGGCAATGACGATCTGAAT-3′  5′-GGTGAGGAGGTACGCGAAG-3′ |
| Mouse *Stfa2l1* | 5′-AGGTCAGACTACTGCTTGAAGA-3′  5′-AGTCCTTGGACGACTTGAACTT-3′ |
| Mouse *Hmgcs2* | 5′-AGAGAGCGATGCAGGAAACTT-3′  5′-AAGGATGCCCACATCTTTTGG-3′ |
| Mouse *Pbld1* | 5′-GACGGCCCCGTATGACTTC-3′  5′-GGACCAGTAACTGCTGAGAAC-3′ |
| Mouse *Ttr* | 5′-CTGCTGTAGACGTGGCTGTAA-3′  5′-CTTCCAGTACGATTTGGTGTCC-3′ |
| Mouse *Itih3* | 5′-TCGAGCCACAGGGCATTAG-3′  5′-GCATGAACGCTGCTGGTCTA-3′ |
| Mouse *Chrna2* | 5′-ACCGCCTGTTCAAACACCTC-3′  5′-CCACATCTATGAGCTGTGCAA-3′ |
| Mouse β-actin | 5′-CCGTGAAAAGATGACCCAGA-3′  5′-TACGACCAGAGGCATACAG-3′ |

**Table S3. Primers for Real-time quantitative PCR analysis in cells**.

| **Gene name** | **Sequences** |
| --- | --- |
| Rat *Decr1* | 5′-GTTTAGACTTGCACTCCTGTGT-3′  5′- AAGCTCTTGAGCCCATGCTT-3′ |
| Rat *nppa* | 5′-GGCCTTTTGGCTCCCAGGCC-3′  5′-CTAAGTGCCGCCCCCGCTTC-3′ |
| Rat *nppb* | 5′-TTGGGCAGAAGATAGACCGGAT-3′  5′-GGTCTTCCTAAAACAACCTCA-3′ |
| Rat *myh7* | 5'-GCAGACAGAGAATGGGGAGCTGTCC-3'  5'-TCGCAATCATGCCGGGCTGAC-3' |
| Rat *β-actin* | 5′-AAGTCCCTCACCCTCCCAAAAG-3′  5′-AAGCAATGCTGTCACCTTCCC-3′ |

**Table S4**. **Compound information used for screening experiments.**

| **Category** | **No.** | **Compounds** |
| --- | --- | --- |
| Benzimidazoles | 1 | 5,6-Dimethylbenzimidazole |
| Carboxylic acids | 2 | 1-Naphthaleneacetic acid |
|  | 3 | 10-Hydroxydecanoic Acid |
|  | 4 | D-(+)-Galacturonic acid |
|  | 5 | Mandelic acid |
|  | 6 | Indole-3-acetic acid |
|  | 7 | Oxalic acid |
|  | 8 | 4-Methoxysalicylic acid |
| Curcuminoid | 9 | Bisdemethoxycurcumin |
| Flavonoids | 10 | Oroxin A |
|  | 11 | Procyanidin B1 |
|  | 12 | Hispidulin |
|  | 13 | 4',6,7-Trimethoxyisoflavone |
|  | 14 | Rhamnocitrin |
|  | 15 | Hamaudol |
|  | 16 | Gossypin |
|  | 17 | Dihydrodaidzein |
|  | 18 | Aromadendrin |
|  | 19 | 6,7,4'-Trihydroxyisoflavone |
|  | 20 | 6-Methoxyluteolin |
|  | 21 | 8-Prenylnaringenin |
|  | 22 | Sakuranetin |
|  | 23 | Skullcapflavone II |
|  | 24 | 4',5,7-Trimethoxyflavone |
|  | 25 | (±)-Catechin hydrate |
|  | 26 | 4'-METHOXYFLAVONE |
|  | 27 | 3,4-Dihydroxyflavone |
|  | 28 | 5,7,3',4'-Tetramethoxyflavone |
|  | 29 | Norwogonin |
|  | 30 | Kaempferol 3-gentiobioside |
|  | 31 | Visnagin |
|  | 32 | 5-Hydroxyflavone |
|  | 33 | Aloeresin D |
|  | 34 | EGCG Octaacetate |
|  | 35 | Tilianin |
|  | 36 | 6-Demethoxytangeretin |
|  | 37 | Quercetagetin |
|  | 38 | 4-METHOXYCHALCONE |
|  | 39 | 7-Hydroxyflavanone |
|  | 40 | 3-Methoxyflavone |
|  | 41 | 4-Hydroxyflavanone |
|  | 42 | 2-Hydroxyflavanone |
|  | 43 | 3,6-Dihydroxyflavone |
|  | 44 | 2-Hydroxychalcone |
|  | 45 | 4'-Hydroxychalcone |
|  | 46 | 7-Methoxyflavone |
|  | 47 | Reynoutrin |
|  | 48 | Theaflavin |
|  | 49 | 7-Hydroxyflavone |
|  | 50 | Procyanidin B2 |
|  | 51 | Apigenin-7-glucuronide |
|  | 52 | Sophoraflavanone G |
|  | 53 | Kurarinone |
|  | 54 | Kaempferol 3-glucorhamnoside |
|  | 55 | MOSLOFLAVONE |
|  | 56 | 5,7-DIMETHOXYFLAVONE |
|  | 57 | 5-hydroxy-7,8-dimethoxyflavone |
|  | 58 | Vitexia-glucoside |
|  | 59 | 5-DEMETHYLNOBILETIN |
|  | 60 | 2''-O-β-L-Galorientin |
|  | 61 | GALANGIN-3-METHYLETHER |
|  | 62 | 4',7-DIMETHOXY-5-HYDROXYFLAVONE |
|  | 63 | Maltol |
|  | 64 | Cyanidin Chloride |
|  | 65 | 3’- Methoxy Puerarin |
|  | 66 | Trifolirhizin |
|  | 67 | Quercetin-3-O-β-D-glucose-7-O-β-D-gentiobiosiden |
|  | 68 | HOMOPLANTAGININ |
|  | 69 | Isoliquiritin apioside |
|  | 70 | 7,2'-dihydroxy-3',4'-dimethoxyisoflavane-7-O-glucoside |
|  | 71 | Karanjin |
|  | 72 | 4-Hydroxycoumarin |
|  | 73 | Quercetagitrin |
|  | 74 | Procyanidin C1 |
|  | 75 | Hesperetin 7-O-glucoside |
|  | 76 | Iristectorigenin A |
|  | 77 | Isorhamnetin-3-O-glucoside |
|  | 78 | Quercimeritrin |
|  | 79 | Luteolin-3-O-beta-D-glucuronide |
|  | 80 | Neoeriocitrin |
|  | 81 | Taxifolin 7-O-rhamnoside |
|  | 82 | Vicenin 3 |
|  | 83 | Vaccarin |
| Glycerolipids | 84 | Glycerol Tri-n-octanoate |
|  | 85 | Glycerol Trieicosanoate |
|  | 86 | Glycerol Tridecanoate |
|  | 87 | Glycerol trilinoleate |
|  | 88 | 1-Oleoyl-rac-glycerol |
| Phenols | 89 | Cannabidiol |
|  | 90 | Hexahydrocurcumin |
|  | 91 | Dimethylcurcumin |
|  | 92 | Chebulinic acid |
|  | 93 | Chebulagic acid |
|  | 94 | 2',3'-Dihydroxy-4'-methoxyacetophenone |
|  | 95 | Homovanillyl alcohol |
|  | 96 | Homogentisic acid |
|  | 97 | Eugenin |
|  | 98 | Atranorin |
|  | 99 | Isopsoralenoside |
|  | 100 | Psoralenoside |
|  | 101 | Pyromeconic acid |
|  | 102 | Gigantol |
|  | 103 | Chicoric Acid |
|  | 104 | Nordihydroguaiaretic acid |
|  | 105 | Deoxyrhapontin |
|  | 106 | 2-5-dihydroxyacetophenone |
|  | 107 | Glucosyringic acid |
|  | 108 | Phenylacetaldehyde |
|  | 109 | 2'-Hydroxyacetophenone |
|  | 110 | BENZYLACETONE |
|  | 111 | trans-Benzylideneacetone |
|  | 112 | Atraric acid |
|  | 113 | 4-Methoxybenzoic acid |
|  | 114 | Forsythoside I |
|  | 115 | Raspberry ketone glucoside |
|  | 116 | 2-HYDROXY-3,4-DIMETHOXYBENZOIC ACID |
|  | 117 | androsin |
|  | 118 | Hydroxytyrosol Acetate |
|  | 119 | Cannabidivarin |
|  | 120 | Gallic aldehyde |
|  | 121 | Anisic aldehyde |
|  | 122 | 2-methoxycinnamaldehyde |
|  | 123 | 3,5-Dimethoxyphenol |
|  | 124 | 6-paradol |
|  | 125 | Gnetol |
|  | 126 | Ginkgolic acid C15:1 |
|  | 127 | Ginkgolic acid C13:0 |
|  | 128 | Ginkgolic acid C17:1 |
|  | 129 | Geraniin |
|  | 130 | Curculigoside |
|  | 131 | 6-Shogaol |
|  | 132 | 8-Gingerol |
|  | 133 | Paeonolide |
|  | 134 | Mulberroside A |
|  | 135 | Vanillin |
|  | 136 | Gossypol |
|  | 137 | Acetovanillone |
|  | 138 | D-DELTA-TOCOPHEROL |
|  | 139 | Orcinol gentiobioside |
|  | 140 | Apiopaeonoside |
|  | 141 | Desmethoxy yangonin |
|  | 142 | Rhaponiticin |
|  | 143 | 4-Ethylphenol |
|  | 144 | Isovanillic acid |
|  | 145 | 4'-Methoxyresveratrol |
|  | 146 | Methylnissolin-3-O-glucoside |
|  | 147 | Acetyl-trans-resveratrol |
|  | 148 | 3,4-Dimethoxybenzaldehyde |
|  | 149 | Zearalenone |
|  | 150 | 4-Hydroxymandelic acid |
|  | 151 | DL -3,4-Dihydroxymandelic acid |
|  | 152 | Agrimol B |
|  | 153 | Dryocrassin ABBA |
|  | 154 | Alnustone |
|  | 155 | DL-Normetanephrine hydrochloride |
|  | 156 | alpha-Arbutin |
|  | 157 | 3,4-Dihydroxyphenylacetic acid |
|  | 158 | 3-Hydroxyphenylacetic acid |
|  | 159 | 4-Methylcatechol |
|  | 160 | 3-Methoxytyramine hydrochloride |
|  | 161 | L-KAWAIN |
|  | 162 | Kakuol |
|  | 163 | Dendrophenol |
|  | 164 | Oxyresveratrol |
|  | 165 | yangonin |
|  | 166 | Thymol |
|  | 167 | Homovanillic acid |
|  | 168 | Punicalagin |
|  | 169 | 10-Gingerol |
|  | 170 | Erianin |
|  | 171 | 7,2'-Dihydroxy-3',4'-dimethoxyisoflavan |
|  | 172 | Pinosylvin |
|  | 173 | Dihydroresveratrol |
|  | 174 | Isorhapontigenin |
|  | 175 | Corilagin |
|  | 176 | 1,2,3,4,6-O-Pentagalloylglucose |
|  | 177 | Rhapontigenin |
|  | 178 | 2'-Hydroxy-5'-methoxyacetophenone |
|  | 179 | 2,6-Dimethoxybenzoic acid |
|  | 180 | Pinostilbene |
|  | 181 | 4-Hydroxybenzyl alcohol |
|  | 182 | Veratric acid |
|  | 183 | Olivetol |
|  | 184 | Tetrahydro Curcumin |
|  | 185 | Methyl gallate |
|  | 186 | Ethyl gallate |
|  | 187 | Methylparaben |
|  | 188 | Methyl syringate |
|  | 189 | β-thujaplicin |
|  | 190 | Sesamol |
|  | 191 | Helicid |
|  | 192 | 3,4-Dihydroxyphenylethanol |
|  | 193 | 6-Gingerol |
|  | 194 | Bakuchiol |
|  | 195 | Protocatechualdehyde |
|  | 196 | Honokiol |
|  | 197 | p-Hydroxybenzaldehyde |
|  | 198 | 5-Hydroxy-1,7-diphenyl-6-hepten-3-one |
|  | 199 | Isoeugenol |
|  | 200 | Chrysophanic Acid |
|  | 201 | Cardamonin |
|  | 202 | 4-Hydroxybenzoic acid |
|  | 203 | (-)-Epigallocatechin Gallate |
|  | 204 | Epigallocatechin |
|  | 205 | Xanthoxyline |
|  | 206 | Sodium Danshensu |
|  | 207 | 3,4,5-Trimethoxyphenol |
|  | 208 | Ethyl Vanillate |
|  | 209 | Paeonol |
|  | 210 | Pterostilbene |
|  | 211 | Phloretic acid |
|  | 212 | Tyrosol |
|  | 213 | Gentisic acid |
|  | 214 | Phloracetophenone |
|  | 215 | Orsellinic acid |
|  | 216 | Morin |
|  | 217 | Ethyl ferulate |
|  | 218 | Caffeic Acid |
|  | 219 | 7-Methoxy-4-methylcoumarin |
|  | 220 | Orsellinic acid ethyl ester |
|  | 221 | (+)-Catechin Hydrate |
|  | 222 | Orcinol glucoside |
|  | 223 | Rosmarinic acid |
|  | 224 | Gossypol acetic acid |
|  | 225 | Salvianolic acid B |
|  | 226 | Methylarbutin |
|  | 227 | Hematoxylin |
|  | 228 | Vitamin E |
|  | 229 | Resveratrol |
|  | 230 | Methyl protocatechuate |
|  | 231 | Terphenyllin |
|  | 232 | Guaiacol |
|  | 233 | Eugenol |
|  | 234 | Vitamin E Acetate |
|  | 235 | 3-Hydroxy-4-methoxyacetophenone |
|  | 236 | Gallic acid |
|  | 237 | Ethylparaben |
|  | 238 | Benzoic acid |
|  | 239 | Cianidanol |
|  | 240 | Tannic acid |
|  | 241 | 3,4-Dimethoxybenzyl alcohol |
|  | 242 | Gallic acid trimethyl ether |
|  | 243 | Methyl EudesMate |
|  | 244 | Protocatechuic acid |
|  | 245 | Ellagic acid |
|  | 246 | Phenylephrine hydrochloride |
|  | 247 | Methyl salicylate |
|  | 248 | Salicylamide |
|  | 249 | Methylsyringol |
|  | 250 | 4-Hydroxyphenylacetonitrile |
|  | 251 | 2'-Hydroxy-4'-methylacetophenone |
|  | 252 | Ethyl 4-hydroxyphenylacetate |
|  | 253 | 4-(4-Methoxyphenyl)-2-butanone |
|  | 254 | Methyl 4-hydroxycinnamate |
|  | 255 | Rubrofusarin-6-O-beta-D-gentiobioside |
|  | 256 | Ethyl salicylate |

**Table S5. The body weight, biochemical characteristics and echocardiographic data in each group.**

| Parameters | ShCon | ShDecr1 | T2D+ShCon | T2D+ ShDecr1 |
| --- | --- | --- | --- | --- |
| FBG (mM/L) | 5.8±0.7 | 5.9±0.6 | 25.3±3.2* | 26.1±2.8* |
| Insulin (mIU/L) | 15.2±2.7 | 15.9±2.8 | 26.8±3.1* | 27.1±3.2* |
| LDL (mM/L) | 0.23±0.02 | 0.24±0.03 | 1.12±0.11* | 1.14±0.13* |
| Total cholesterol (mM/L) | 2.2±0.3 | 2.3±0.5 | 4.8±0.6* | 4.7±0.3* |
| Triacylglycerols (mM/L) | 1.5±0.4 | 1.6±0.3 | 3.7±0.4* | 3.9±0.5* |
| BW (g) | 30.7±1.2 | 31.2±1.3 | 28.2±1.4 | 29.1±1.6 |
| HW (g) | 0.14±0.03 | 0.13±0.04 | 0.22±0.06* | 0.16±0.05† |
| HW/BW (mg/g) | 4.11±0.87 | 4.16±0.78 | 7.12±0.98* | 5.12±0.62† |
| HW/TL (g/cm) | 0.08±0.02 | 0.08±0.04 | 0.14±0.05* | 0.10±0.03† |
| LVESD (mm) | 1.77±0.23 | 1.78±0.28 | 2.21±0.37* | 1.83±0.21† |
| LVEDD (mm) | 3.46±0.27 | 3.42±0.31 | 3.52±0.38 | 3.43±0.41 |
| LVAWs (mm) | 1.52±0.12 | 1.54±0.15 | 2.08±0.12* | 1.61±0.14† |
| LVAWd (mm) | 0.95±0.11 | 0.98±0.13 | 1.45±0.18* | 1.08±0.15† |
| LVPWs (mm) | 1.44±0.16 | 1.45±0.15 | 1.67±0.17* | 1.46±0.12† |
| LVPWd (mm) | 0.92±0.09 | 0.93±0.07 | 1.21±0.09* | 0.98±0.07† |

Notes: FBG, fasting blood glucose; LDL, low density lipoprotein; BW, body weight; HW, heart weight; TL, tibia length; LV, left ventricular; LVESD, LV end systolic dimension; LVEDD, LV end diastolic dimension; LVAW, LV anterior wall thickness; LVPW, LV posterior wall thickness.

**Table S6. The body weight, biochemical characteristics and echocardiographic data in each group.**

| Parameters | Vector | Decr1 OE | T2D+Vector | T2D+Decr1 OE |
| --- | --- | --- | --- | --- |
| FBG (mM/L) | 5.9±0.5 | 5.7±0.4 | 24.9±3.3* | 25.2±2.9* |
| Insulin (mIU/L) | 14.7±2.1 | 15.1±2.3 | 26.1±3.4* | 25.7±3.1* |
| LDL (mM/L) | 0.25±0.04 | 0.23±0.05 | 1.01±0.13* | 1.11±0.14* |
| Total cholesterol (mM/L) | 2.3±0.4 | 2.2±0.3 | 4.6±0.4* | 4.8±0.5* |
| Triacylglycerols (mM/L) | 1.6±0.2 | 1.5±0.5 | 3.6±0.3* | 3.8±0.6* |
| BW (g) | 31.1±1.2 | 30.8±1.1 | 28.4±1.5 | 28.1±1.3 |
| HW (g) | 0.15±0.04 | 0.14±0.03 | 0.21±0.07* | 0.27±0.06† |
| HW/BW (mg/g) | 4.23±0.81 | 4.19±0.71 | 7.23±0.65* | 8.92±0.72† |
| HW/TL (g/cm) | 0.08±0.03 | 0.08±0.06 | 0.15±0.04* | 0.22±0.05† |
| LVESD (mm) | 1.74±0.21 | 1.75±0.24 | 2.20±0.25* | 2.85±0.22† |
| LVEDD (mm) | 3.35±0.24 | 3.37±0.23 | 3.46±0.35 | 3.49±0.43 |
| LVAWs (mm) | 1.51±0.13 | 1.52±0.11 | 2.05±0.13* | 2.65±0.15† |
| LVAWd (mm) | 0.93±0.12 | 0.93±0.09 | 1.41±0.11* | 1.88±0.12† |
| LVPWs (mm) | 1.38±0.12 | 1.39±0.11 | 1.68±0.13* | 1.96±0.11† |
| LVPWd (mm) | 0.91±0.06 | 0.92±0.07 | 1.22±0.07* | 1.68±0.09† |

Notes: FBG, fasting blood glucose; LDL, low density lipoprotein;BW, body weight; HW, heart weight; TL, tibia length; LV, left ventricular; LVESD, LV end systolic dimension; LVEDD, LV end diastolic dimension; LVAW, LV anterior wall thickness; LVPW, LV posterior wall thickness.

**Table S7. The body weight, biochemical characteristics and echocardiographic data in each group.**

| Parameters | T2D+ShCon | T2D+ShDecr1 | T2D+ShDecr1+PDK4 OE |
| --- | --- | --- | --- |
| FBG (mM/L) | 25.1±2.5 | 26.1±2.7 | 25.5±2.2* |
| Insulin (mIU/L) | 27.1±2.4 | 24.9±2.2 | 24.1±3.3* |
| LDL (mM/L) | 1.11±0.03 | 1.19±0.02 | 1.13±0.05* |
| Total cholesterol (mM/L) | 4.6±0.5 | 4.9±0.4 | 4.9±0.7* |
| Triacylglycerols (mM/L) | 3.6±0.5 | 3.5±0.3 | 3.9±0.4* |
| BW (g) | 28.2±1.3 | 27.9±1.4 | 28.1±1.5 |
| HW (g) | 0.23±0.07 | 0.15±0.06* | 0.22±0.07† |
| HW/BW (mg/g) | 7.22±0.81 | 4.45±0.73* | 6.98±0.75† |
| HW/TL (g/cm) | 0.15±0.03 | 0.09±0.05* | 0.14±0.06† |
| LVESD (mm) | 2.23±0.35 | 1.79±0.23* | 2.19±0.29† |
| LVEDD (mm) | 3.54±0.29 | 3.46±0.33 | 3.56±0.34 |
| LVAWs (mm) | 2.12±0.11 | 1.58±0.08* | 2.05±0.13† |
| LVAWd (mm) | 1.47±0.12 | 0.99±0.14* | 1.43±0.13† |
| LVPWs (mm) | 1.68±0.15 | 1.46±0.12* | 1.65±0.14† |
| LVPWd (mm) | 1.23±0.07 | 0.97±0.08* | 1.22±0.08† |

Notes: FBG, fasting blood glucose; LDL, low density lipoprotein; BW, body weight; HW, heart weight; TL, tibia length; LV, left ventricular; LVESD, LV end systolic dimension; LVEDD, LV end diastolic dimension; LVAW, LV anterior wall thickness; LVPW, LV posterior wall thickness.

**Table S8. The body weight, biochemical characteristics and echocardiographic data in each group.**

| Parameters | Con | T2D | T2D+Atranorin | T2D+Kurarinone |
| --- | --- | --- | --- | --- |
| FBG (mM/L) | 5.6±0.4 | 26.1±2.5 | 26.3±3.1* | 26.5±3.1* |
| Insulin (mIU/L) | 14.1±2.4 | 25.1±2.9 | 26.1±3.2* | 24.3±3.1* |
| LDL (mM/L) | 0.23±0.03 | 1.14±0.13 | 1.14±0.12* | 1.11±0.14* |
| Total cholesterol (mM/L) | 2.3±0.33 | 4.9±0.4 | 4.8±0.6* | 4.6±0.4* |
| Triacylglycerols (mM/L) | 1.6±0.3 | 3.8±0.4 | 3.9±0.3* | 3.7±0.5* |
| BW (g) | 30.8±1.4 | 28.2±1.5* | 28.7±1.6* | 28.4±1.5* |
| HW (g) | 0.15±0.04 | 0.23±0.05* | 0.17±0.07† | 0.16±0.06† |
| HW/BW (mg/g) | 4.34±0.53 | 7.26±0.78* | 4.56±0.51† | 4.71±0.53† |
| HW/TL (g/cm) | 0.08±0.03 | 0.15±0.06* | 0.11±0.07† | 0.09±0.05† |
| LVESD (mm) | 1.76±0.21 | 2.23±0.24 | 1.79±0.21* | 1.81±0.23† |
| LVEDD (mm) | 3.31±0.39 | 3.52±0.35 | 3.49±0.33 | 3.61±0.43 |
| LVAWs (mm) | 1.48±0.11 | 2.11±0.18* | 1.58±0.15† | 1.62±0.16† |
| LVAWd (mm) | 0.94±0.12 | 1.46±0.14* | 0.97±0.17† | 1.01±0.16† |
| LVPWs (mm) | 1.43±0.14 | 1.68±0.15* | 1.47±0.14† | 1.45±0.16† |
| LVPWd (mm) | 0.94±0.08 | 1.23±0.09* | 0.96±0.07† | 0.99±0.08† |

Notes: FBG, fasting blood glucose; LDL, low density lipoprotein; BW, body weight; HW, heart weight; TL, tibia length; LV, left ventricular; LVESD, LV end systolic dimension; LVEDD, LV end diastolic dimension; LVAW, LV anterior wall thickness; LVPW, LV posterior wall thickness.
